# Thermoresponsive mixed polymer brush to effectively control the adhesion and separation of stem cells by altering temperature

**DOI:** 10.1016/j.mtbio.2023.100627

**Published:** 2023-04-07

**Authors:** Kenichi Nagase, Haruno Wakayama, Junnosuke Matsuda, Naoto Kojima, Hideko Kanazawa

**Affiliations:** Faculty of Pharmacy, Keio University, 1-5-30 Shibakoen, Minato, Tokyo, 105-8512, Japan

**Keywords:** Thermoresponsive polymer, Polymer brush, Mesenchymal stem cell, Cell separation, Temperature-responsive chromatography

## Abstract

During the last few decades, thermoresponsive materials for modulating cell adhesion have been investigated for the application of tissue engineering. In this study, we developed thermoresponsive mixed polymer brushes consisting of poly(*N*-isopropylacrylamide) (PNIPAAm) and poly(*N,N*-dimethylaminopropylacrylamide) (PDMAPAAm). The mixed polymer brushes were prepared on a glass substrate via the reversible addition-fragmentation chain transfer polymerization of DMAPAAm and subsequent atom transfer radical polymerization of NIPAAm. The mixed polymer brushes grafted to glass exhibited increased cationic properties by increasing the grafted PDMAPAAm length. The shrinking and extension of PNIPAAm exposed and concealed PDMAPAAm, respectively, indicating that the surface cationic properties can be controlled by changing the temperature. At 37 ​°C, the prepared mixed polymer brushes enhanced cell adhesion through their electrostatic interactions with cells. They also exhibited various thermoresponsive adhesion and detachment properties using various types of cells, such as mesenchymal stem cells. Temperature-controlled cell adhesion and detachment behavior differed between cell types. Using the prepared mixed polymer brush, we separated MSCs from adipocytes and HeLa cells by simply changing the temperature. Thus, the thermoresponsive mixed polymer brushes may be used to separate mesenchymal stem cells from their differentiated or contaminant cells by altering the temperature.

## Highlights

• A thermoresponsive cationic mixed polymer brush was prepared.
• The mixed polymer brush exhibits thermoresponsive property.
• Temperature controlled stem cell adhesion on mixed polymer brush was performed.
• Prepared mixed polymer brush separates stem cell from contaminant cells.

## Graphical abstract

**Figure:**
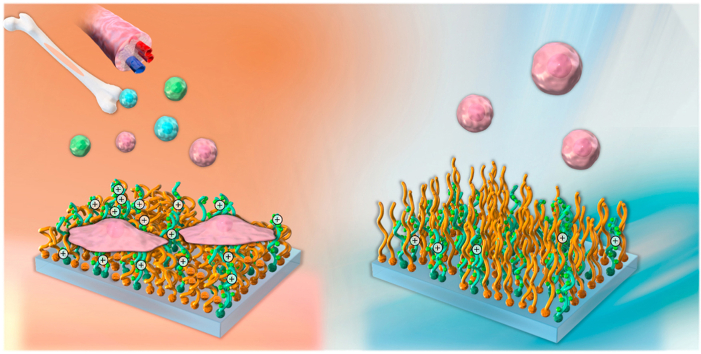

## 1 Introduction

In last few decades, tissue engineering and regenerative therapy using cell transplantation have emerged as effective biomedical treatments [[1], [2], [3], [4], [5], [6]]. For many cell-based tissue engineering and therapy protocols, cell separation is required to prepare cell suspensions or cellular tissues. For instance, in a sample of cells collected from a human, various contaminant cells are mixed with target cells. In stem cell culture, differentiated cells co-exist with stem cells. Therefore, target cells must be purified from contaminant cells before they can be used for treatment or other applications.

Various cell separation methods have been developed and investigated [[7], [8], [9], [10], [11], [12], [13], [14], [15], [16], [17], [18]]. Cell separation methods using fluorescent-labelled antibodies or magnetic particle-conjugated antibodies are widely used and accurate. However, these methods necessitate the labelling of the cell surface with different antibodies, which can alter the biological activity of sorted cells. Thus, cell separation without labelling is advantageous. Cell separation using thermoresponsive polymers is a novel cell separation method that does not require labelling of the cell surface because the interaction between thermoresponsive polymers and target cells can be controlled by changing the temperature. Various types of thermoresponsive polymers, such as poly(*N*-isopropylacrylamide) (PNIPAAm), poly(vinyl methyl ether), poly(*N*-vinylcaprolactam), poly(glycidyl methyl ether-*co*-glycidyl ethyl ether), poly(2-isopropyl-2-oxazoline), and poly[oligo(ethylene glycol) methyl ether methacrylate] (POEGMA), have been investigated for biomedical applications [[19], [20], [21], [22], [23], [24], [25], [26], [27], [28], [29], [30]]. Among these polymers, PNIPAAm has attracted attention because it exhibits temperature-dependent hydrophobic and hydrophilic changes, leading to cell adhesion and detachment.

PNIPAAm-modified cell culture dishes have been investigated for preparing monolayer cellular tissue or cell sheets [23,31]. Various types of cell sheets can be applied to various types of the regenerative medicines [4,5,[32], [33], [34]]. PNIPAAm has been successfully used in functional stem cell culture materials [[35], [36], [37], [38]]. For instance, PNIPAAm copolymer can be coated on cell culture dishes with POEGMA and poly(acrylic acid) copolymers [36]. These dishes can then be used to continuously culture human adipose-derived stem cells (hADSCs) and embryonic stem cells (hESCs) for five and three cycles, respectively [36]. In addition, PNIPAAm copolymers and oligovitronectin-grafted poly(acrylic acid-*co*-styrene) or recombinant vitronectin have been used in cell culture dishes [35]. Such culture dishes can be used to continuously culture human pluripotent stem cells (hPSCs) and maintain their pluripotency [35].

The unique characteristics of PNIPAAm have been investigated for cell separation [39]. PNIPAAm and laminin-coated culture dishes have been used to separate hPSC-differentiated cardiomyocytes from undifferentiated hPSCs; cardiomyocytes selectively detach from the dish because of their relatively weaker adhesion than undifferentiated hPSCs [39].

PNIPAAm brush-modified substrates prepared though atom transfer radical polymerization have also been studied as cell separation materials [[40], [41], [42]]. In the case of the cell separation materials using PNIPAAm, PNIPAAm-grafted substrates, such as glass plates, silica beads, or microfibers, are used. At 37 ​°C, cells adhere to the PNIPAAm-modified substrate because the grafted PNIPAAm on the substrate dehydrates and shrinks. When the temperature is reduced to 20 ​°C, PNIPAAm becomes hydrophilic and swollen, and the adhered cells on PNIPAAm-grafted substrates detach and recover. The target cells selectively adhere to PNIPAAm at 37 ​°C or selectively recover from PNIPAAm at 20 ​°C. Therefore, temperature can influence the adhesion and detachment behavior of cells on PNIPAAm-grafted substrates.

To improve cell selectivity, copolymerization of PNIPAAm has been investigated by incorporating ionic monomers to PNIPAAm via random copolymerization or block copolymerization [[43], [44], [45]]. Compared with the PNIPAAm homopolymer brush, the prepared ionic PNIPAAm copolymer-grafted substrate exhibits a relatively higher adhesion selectivity for mesenchymal stem cells (MSCs). However, the amount of ionic monomer incorporation is limited because of the thermoresponsive property of PNIPAAm [46,47].

To resolve this issue, we developed mixed polymer brush-modified glass substrates composed of thermoresponsive PNIPAAm and cationic poly(*N,N*-dimethylamino propylacrylamide) (PDMAPAAm) as functional cell separating materials. PDMAPAAm is a cationic polymer with a weak base dimethyl amino group. The dimethyl amino group on PDMAPAAm is protonated, conferring it cationic properties [48,49]. PNIPAAm and PDMAPAAm were separately grafted on glass substrates via the combination of atom transfer radical polymerization (ATRP) and reversible addition-fragmentation chain transfer (RAFT) polymerization. The prepared mixed polymer brush was characterized, and the temperature-dependent adhesion and detachment of MSCs to our novel mixed polymer brush were tested to evaluate cell separation performance.

## 2 Materials and methods

### 2.1 Preparation of mixed polymer brush-grafted glass

**Fig. 1:**
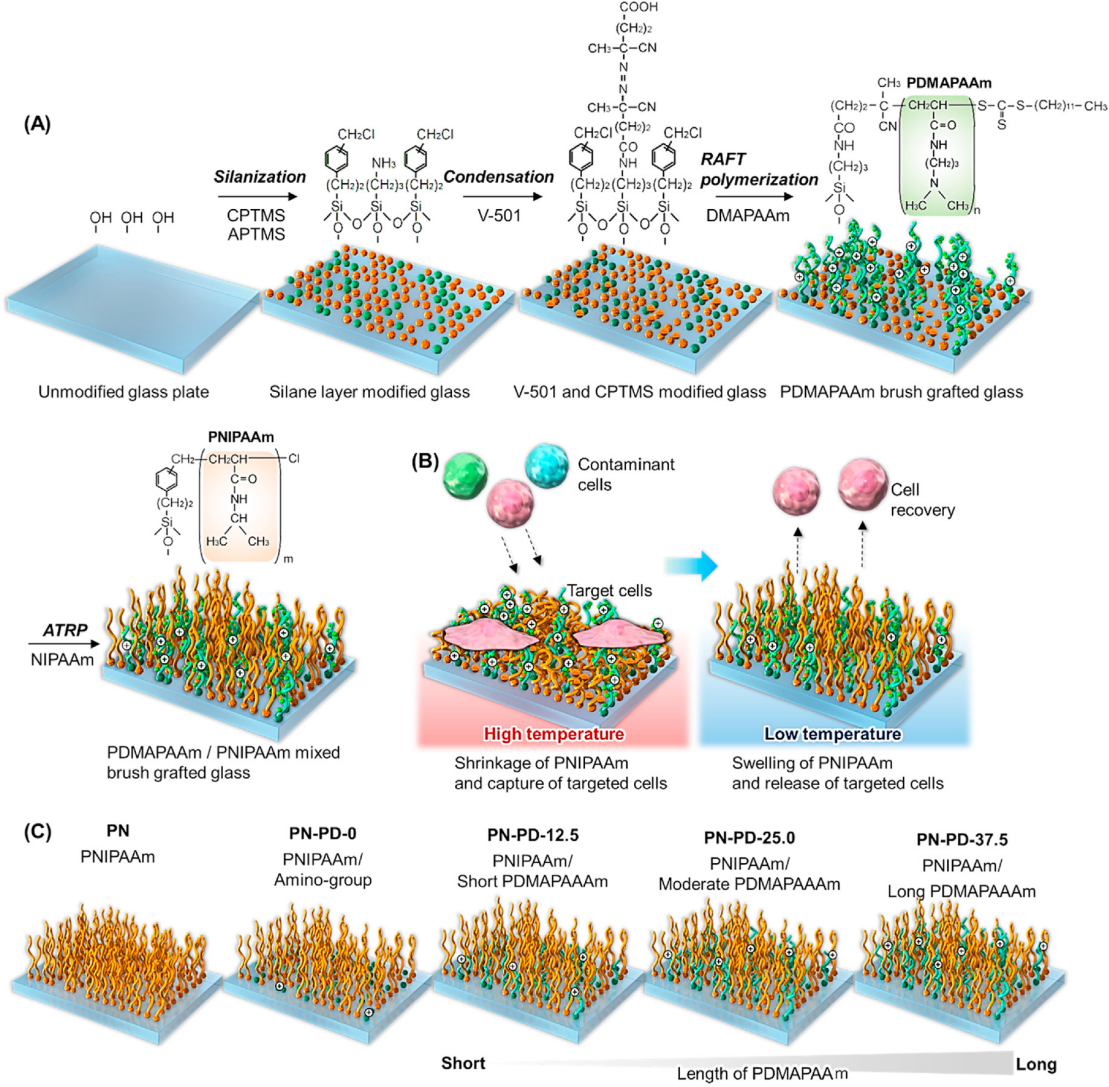
Schematic illustration for the prepared mixed polymer brush for the selective capture of cells. **(A)** Preparation of a mixed polymer brush consisting of PDMAPAAm and PNIPAAm. **(B)** Temperature-controlled cell capture using the prepared mixed polymer brush. **(C)** Prepared mixed polymer brushes.

A thermoresponsive cationic mixed polymer brush was prepared on glass substrates via RAFT polymerization to graft PDMAPAAm and subsequent ATRP to graft PNIPAAm (Fig. 1A). All reagents used for the preparation of the mixed polymer brush are described in the supplementary materials.

Glass plates were set in a glass holder and cleaned using a plasma gas cleaner. The glass plates in the glass holder were set in a flask, and the relative humidity was reduced below 10% by flowing nitrogen gas for 15 ​min. APTMS (0.58 ​mL, 3.28 ​mmol) and CPTMS (2.43 ​mL, 9.64 ​mmol) were added into toluene (300 ​mL). The solution was bubbled with nitrogen for 30 ​min and then added to the glass plates in flask; salinization was conducted at 25 ​°C for 18 ​h. The glass plates were rinsed with toluene and methanol and then dried at 110 ​°C for 3 ​h. To prepare PNIPAAm homopolymer brushes, solely CPTMS modified glass plates were prepared using the CPTMS solution in place of the APTMS and CPTMS mixed solution. CPTMS (3.24 ​mL, 12.9 ​mol) was added in 300 ​mL of toluene, and the glass plates were rinsed with the solution using the previous procedure.

Radical initiator (V-501) was allowed to react with the amino group of APTMS on glass plates via the condensation reaction. Silane layer-modified glass plates were set in a glass holder, and the glass plates were placed in a flask. V-501 (3.17 ​g, 11.3 ​mmol) and EEDQ (5.56 ​g, 22.5 ​mmol) were added in DMF (300 ​mL). The solution was bubbled with nitrogen for 30 ​min and added to the glass plate in the flask. The solution was allowed to react with the glass plates in the flask at 25 ​°C for 20 ​h. After the reaction, the glass plates were rinsed with methanol and dried.

PDMAPAAm was grafted on glass plates through RAFT polymerization. For preparing 12.5 ​mM of DMAPAAm solution, DMAPAAm (0.586 ​g, 3.75 ​mmol), V-501 (1.00 ​mg, 3.56 ​μmol), and 2-(dodecylthiocarbonothioylthio)-2-methylpropionic acid (54.7 ​mg, 0.150 ​mmol) were added in a 1,4-dioxane (300 ​mL) flask. Then, the V-501 immobilized glass plates were immersed in the solution. Nitrogen bubbling of the solution was performed for 45 ​min. The RAFT polymerization was conducted at 70 ​°C for 24 ​h. The glass plates were rinsed with methanol and then dried. During the polymerization, different DMAPAAm concentrations of 12.5, 25.0, and 37.5 ​mM were used to modulate the chain length of PDMAPAAm.

PNIPAAm was grafted on the PDMAPAAm-grafted glass plates via the activators regenerated by electron transfer (ARGET-ATRP). NIPAAm (29.1 ​g, 257 ​mmol) was added in the mixed solvent of 2-propanol (280 ​mL) and pure water (20 ​mL) in a flask. Oxygen in the solution was removed by flowing argon for 30 ​min. Then, l-ascorbic acid (106 ​mg, 0.602 ​mmol) and CuCl_2_ (8.07 ​mg, 0.0600 ​mmol) were dissolved in the reaction solution, and argon gas was flowed for 30 ​min. Then, Me_6_TREN (138 ​mg, 0.599 ​mmol) was dissolved in the solution. The PDMAPAAm-grafted glass plates, contained in the glass holder, were placed in the other flask. The reaction solution and glass plates were placed in a glove bag. Oxygen in the glove bag was taken out by flowing argon gas. The reaction solution was poured onto the glass plates in the flask. α-Chloro-*p*-xylene (26.3 ​μL, 0.198 ​mmol) was dissolved in the solution, and the flask was sealed. ATRP was conducted at 25 ​°C for 16 ​h. The glass plates were washed with methanol in an ultrasonic cleansing bath for 30 ​min. Then, the glass plates were washed using the mixed solvent (methanol: 50 ​mM EDTA aqueous solution (1:1)) in an ultrasonic cleansing bath for 30 ​min. Then, the mixed polymer brush-modified glass was dried in a vacuum.

The prepared mixed polymer brush was characterized through X-ray photoelectron spectroscopy (XPS), zeta potential measurement, ellipsometry, atomic force microscopy, contact angle measurement, and protein adsorption. Grafted polymer length was estimated through ^1^H NMR and gel permeation chromatography (GPC).

Surface elemental composition of the mixed polymer brush was obtained by XPS (Quantera-SXM, ULVAC-PHI, Kanagawa, Japan). The x-ray source was monochromatic Alkα, and the take-off angle was 15°. The zeta potential of the mixed polymer brush was measured using a zeta potential analyzer (ELSZ2KOP, Otsuka Electronics, Osaka, Japan). The thickness of the prepared mixed polymer brush was measured by ellipsometry (RC2-DI, J.A. Woollam, Lincoln, NE, USA). Surface morphology of the mixed polymer brush was observed by atomic force microscopy (SPM9600, Shimadzu, Kyoto). The contact angle of the mixed brush was measured with a contact angle meter (ST-1; Surfgauge Instruments, Matsudo, Japan) using 2 ​μL of pure water droplet. Protein adsorption was conducted by immersing the prepared mixed polymer brushes in protein solutions. Rhodamine-conjugated fibronectin solution (5 ​μg/mL) was prepared in PBS. Fluorescein isothiocyanate bovine serum albumin (5 ​μg/mL) was prepared in PBS. The mixed polymer brush was immersed in the protein solutions at 37 ​°C for 2 ​h. Then, the mixed polymer brush grafted substrates were rinsed with PBS, and the adsorbed protein on the mixed polymer brush was observed by fluorescence microscopy (BZ-X800; Keyence, Osaka, Japan). Luminance of the fluorescent protein adsorbed polymer brushes was analyzed using an image analysis software (ImageJ, NIH, Bethesda, MA, USA). Luminance ratio was estimated by the ratio of the luminance of the mixed polymer brush to that of PN. The zeta potentials of the mixed polymer brush before and after incubation with cell culture medium were observed to investigate the effect of protein adsorption on the electrostatic properties of the mixed polymer brush. The prepared mixed polymer brush was immersed in Dulbecco's modified eagle medium (DMEM) supplemented with 10% FBS at 37 ​°C for 2 ​h and then rinsed with PBS and water. The zeta potential of the mixed polymer brush was observed using a zeta potential analyzer (Surpass3, Anton-Paar, Graz, Austria).

The prepared PDMAPAAm in the reaction solution through RAFT polymerization was obtained to determine the molecular weight of grafted PDMAPAAm. 1,4-Dioxane was evaporated, and the residue was dissolved in methanol. The solution was dialyzed using dialysis membrane (MWCO:1000) against pure water for 5 days, and the water was changed every day. The polymer solution was freeze dried, and a purified PDMAPAAm was obtained. The molecular weight of PDMAPAAm was estimated by ^1^H NMR (UI1500 Model 400 Unity Inova, Varian, CA, USA) using chloroform-D1 as a solvent. The prepared PNIPAAm in ATRP was obtained to determine the molecular weight of the grafted PNIPAAm. The reaction solution was dialyzed using a dialysis membrane tube (MWCO:1000) against EDTA aqueous solution for 1 day and then against water for 4 days, with the water changed every day. Finally, PNIPAAm solution was freeze dried, and a purified PNIPAAm was obtained. The molecular weight of PNIPAAm was obtained by GPC (HLC-8020GPC, Tosoh, Tokyo, Japan) using DMF containing 50 ​mM LiCl.

### 2.2 Temperature-controlled selective cell adhesion and detachment

Nine types of cells were tested to investigate the cell adhesion and detachment properties of the prepared mixed polymer brush. Supplementary materials contain detailed information about the cells, such as the company, cell culture reagents used, and cell culture method for each cell (Tables S1 and S2).

The mixed polymer brush-grafted glass was divided into two 24 ​mm ​× ​25 ​mm pieces, which were placed into 35 ​mm-diameter polystyrene dishes. The dishes were sterilized by ethanol in a clean bench and then rinsed twice with PBS. Cell suspensions (5 ​× ​10^4^ ​cells/mL) were prepared using culture medium, and the cell suspension (2 ​mL) was seeded to the dishes. The dishes were incubated at 37 ​°C for 6 or 24 ​h. Subsequently, the dishes were incubated at 20 ​°C for 4 ​h. During the incubation at 37 ​°C and 20 ​°C, the dishes were observed using a phase contrast microscope (CKX53, Olympus, Tokyo) at a predetermined period. Cell adhering ratio (%) was obtained by calculating the percentage of adhered cells to the seeded cells in the obtained phase contrast images. The recovery ratio at 37 ​°C was obtained by subtracting the adhering ratio at 37 ​°C from the total seeded cells (100%), whereas the recovery ratio at 20 ​°C was obtained by subtracting of the adhering ratio at 20 ​°C from that at 37 ​°C.

In the cell separation experiment, adipocyte-BM was stained red using cell staining reagent. The stained adipocyte-BM and BMMSCs were mixed at a ratio of 1:1. Then, 2 ​mL of cell suspension (5 ​× ​10^4^ ​cells/mL) was seeded on the mixed polymer brush-grafted glass plate (PN-PD-37.5) in a dish. The dish was incubated at 37 ​°C for 6 ​h in a CO_2_ incubator. After incubation, cell adhesion on copolymer-modified glass was observed by fluorescence microscopy (BZ-X800; Keyence). The cell adhesion ratio was defined as the ratio of adhered cells in the microscopic images to seeded cells. Then, the dish was rinsed with culture medium at 37 ​°C, followed by the addition of culture medium cooled to 20 ​°C. The dish was incubated at 20 ​°C for 3 ​h in a CO_2_ incubator. Cell adhesion on the glass was observed by fluorescence microscopy (BZ-X800). The recovered cells were evaluated by subtracting the adhered cell ratio at 20 ​°C from that at 37 ​°C. The recovered cell composition was calculating by dividing the recovered cell ratio for each type of cell by the total recovered cell ratio for the two types of cells.

## 3 Results and discussion

### 3.1 Characterization of the prepared thermoresponsive mixed polymer brush

**Fig. 2:**
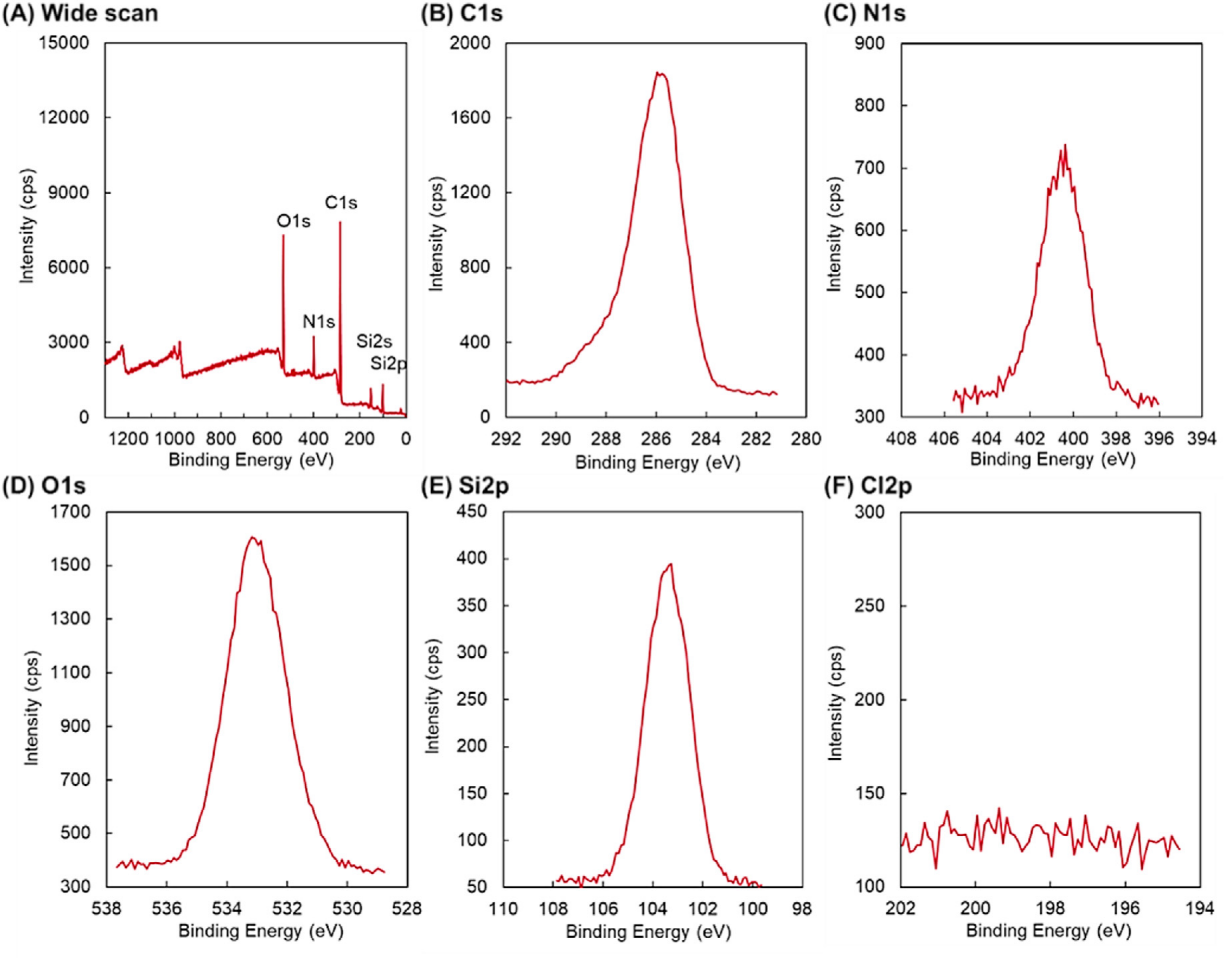
XPS spectrum of PDMAPAAm/PNIPAAm mixed brush-grafted glass substrate (PN-PD-37.5). Take-off angle of 15°. XPS spectrum of PDMAPAAm/PNIPAAm mixed brush grafted glass substrates (PN, PN-PD-0, PN-PD-12.5, and PN-PD-25.0) are shown in supplementary materials.

**Fig. 3:**
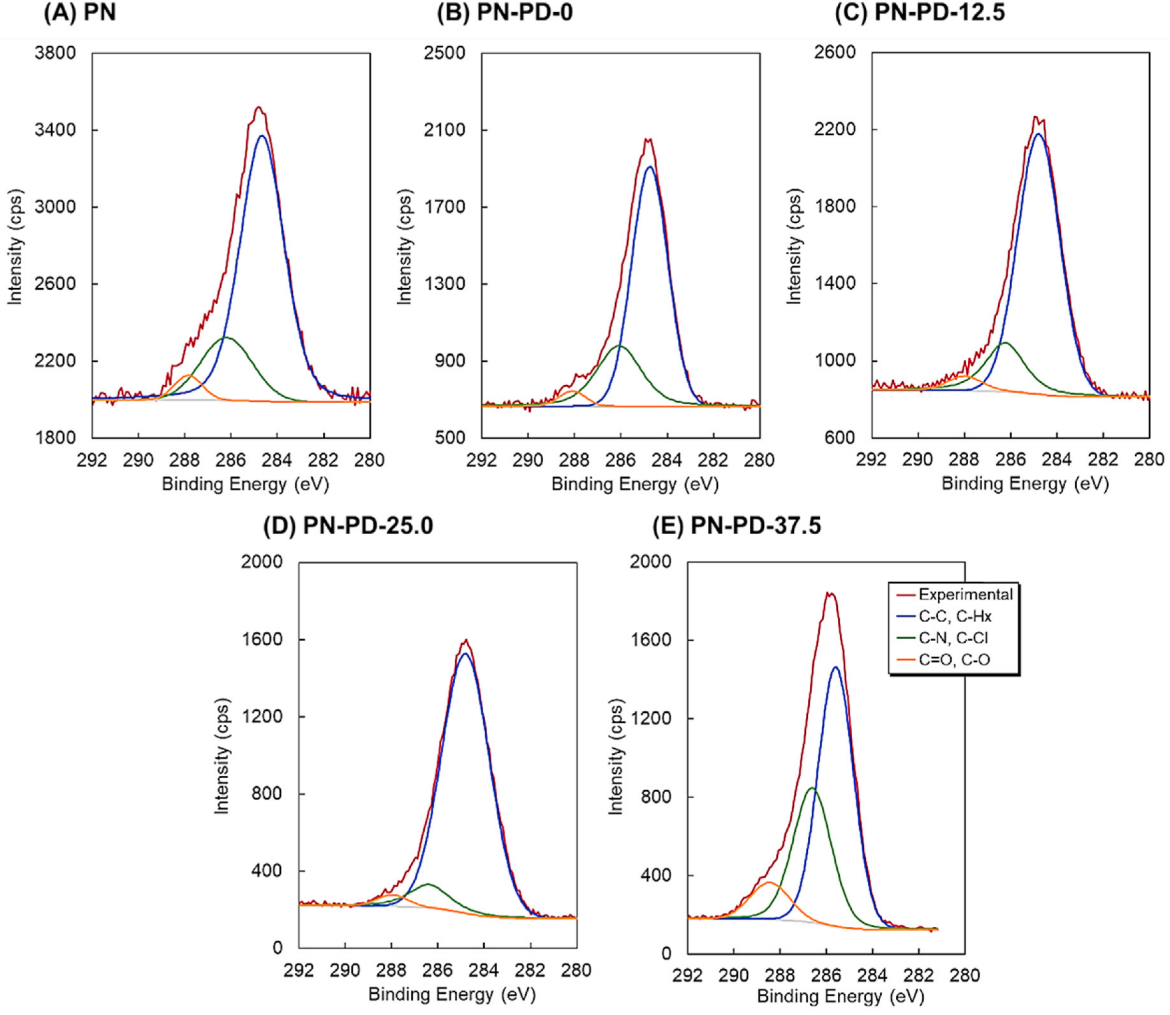
C1s peak deconvolution of PDMAPAAm/PNIPAAm mixed brush grafted glass substrate. Take-off angle of 15°.

**Fig. 4:**
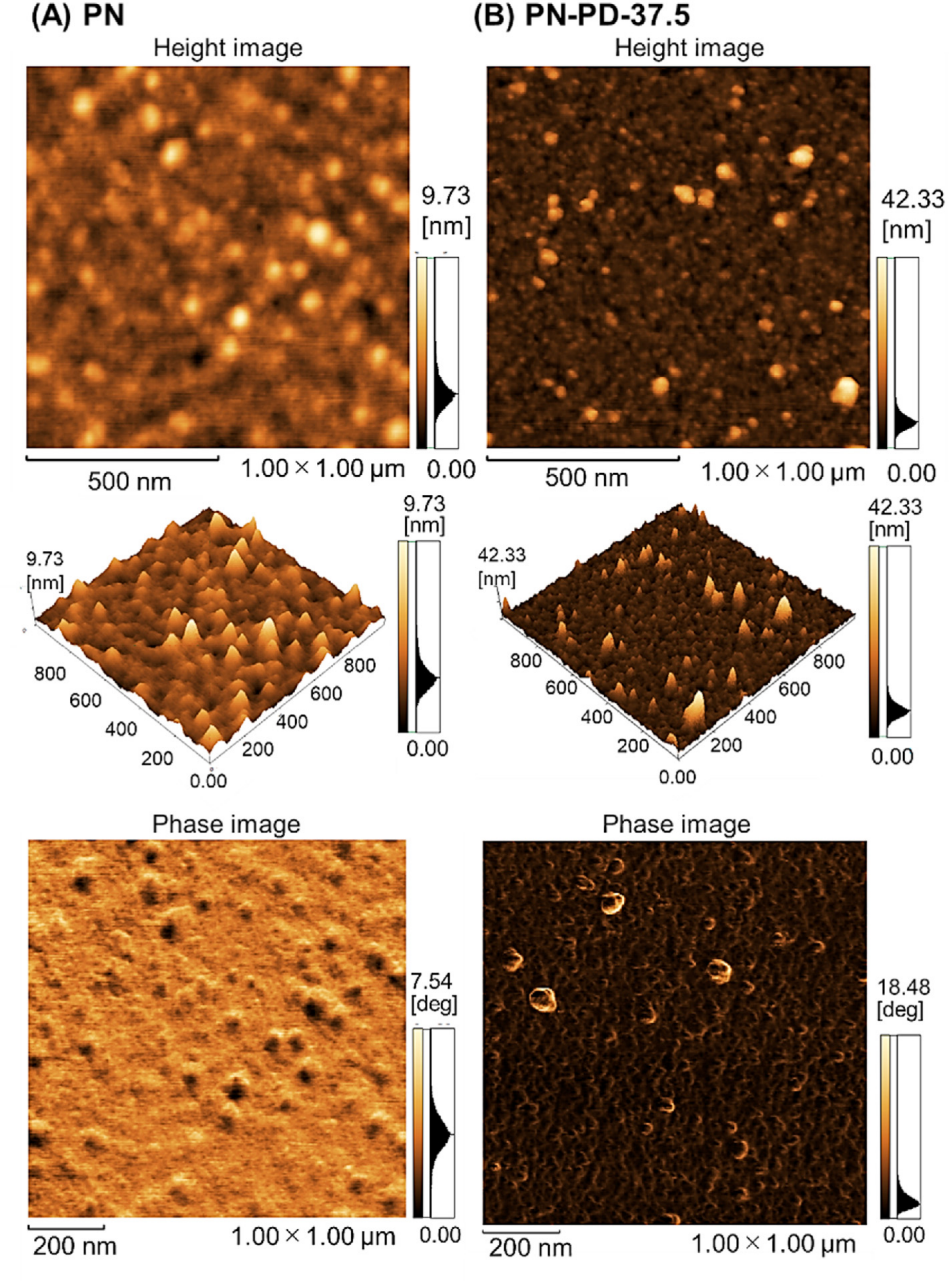
Surface morphology of the prepared mixed polymer brush observed by AFM.

A mixed thermoresponsive and cationic polymer brush was prepared using RAFT polymerization and ARGET-ATRP (Fig. 1). The prepared mixed polymer brush was characterized by XPS (Fig. 2, Fig. 3, Figs. S2–S5, and Table 1), GPC (Table 2), ellipsometry (Fig. S6), AFM (Fig. 4), zeta potential (Table 2), contact angle (Table 2), and protein adsorption (Fig. S7). The surface elemental composition of the prepared mixed polymer brush-modified glass was observed using XPS (Fig. 2, Fig. 3, Figs. S2–S5, and Table 1). The carbon of the grafted PNIPAAm and PDMAPAAm on glass was detected on all the prepared mixed polymer brush-grafted substrates. PN exhibited a relatively higher carbon composition than PN-PD-0, PN-PD-12.5, PN-PD-25.0, and PN-PD-37.5. This result is attributed to the difference in the graft density of the polymer. PN was prepared through silane coupling reaction with only CPTMS, followed by the ATRP of NIPAAm. Thus, densely packed PNIPAAm brush was grafted on PN. By contrast, mixed polymer brushes PN-PD-0, PN-PD-12.5, PN-PD-25.0, and PN-PD-37.5 were prepared through the silane coupling reaction using 75% of CPTMS and 25% of APTMS mixed reaction solution, followed by the RAFT polymerization of DMAPAAm and ATRP of NIPAAm. Thus, the grating density of PNIPAAm was approximately 75% that of PN. Although PDMAPAAm was also grafted on the mixed polymer brushes with PNIPAAm, the graft density of PDMAPAAm through RAFT polymerization was lower than that of PNIPAAm through ATRP. Thus, the polymer densities of the mixed polymer brushes were relatively lower than that of the homopolymer brush PN, which caused the relatively lower carbon composition of the mixed polymer brushes. The silicon of the glass substrate was detected by XPS, which revealed a low silicon composition. The silicon-to-oxygen ratio of the mixed polymer brush was 0.370–0.468, which was smaller than that of the glass substrate (0.500). This is because the oxygen contained PNIPAAm and PDMAPAAm was detected by XPS, leading to increased oxygen composition and consequently decreased Si/O ratio. Chloride was not detected in the mixed polymer brushes. A previous report demonstrated that the terminal chloride of polymer brush is either buried inside the polymer brush or lost during the long polymerization of ATRP [50]. Similarly, the terminal chloride of the mixed polymer brushes was not detected by XPS. C1s peak deconvolution was conducted to estimate the composition of the bonds (Fig. 3). Peak at 288 ​eV was observed in the deconvoluted peak because of the carbonyl bond of PNIPAAm and PDMAPAAm. The result indicated that PNIPAAm and PDMAPAAm were successfully modified on the glass substrate.

**Table 1:** Elemental analyses of mixed polymer brushes grafted glass substrates via X-ray photoelectron spectroscopy at a take-off angle of 15°.

Code	Atom (%)					N/C ratio	Si/O ratio
	C	N	O	Si	Cl		
PN	76.3	12.1	11.6	n.d.	n.d.	0.158	0
PN-PD-0	61.1	7.2	21.6	10.1	n.d.	0.118	0.468
PN-PD-12.5	60.2	7.4	21.5	11.0	n.d.	0.123	0.512
PN-PD-25.0	66.2	8.5	21.5	8.0	n.d.	0.128	0.372
PN-PD-37.5	64.4	9.7	18.9	7.0	n.d.	0.151	0.370
PNIPAAm^a^	75.0	12.5	12.5	–	–	0.167	–
PDMAPAAm^a^	72.7	18.2	9.09	–	–	0.250	–
CPTMS^a^	70.6	–	17.6	5.88	5.88	–	0.333
glass substrate^a^	–	–	66.7	33.3	–	–	0.500

a Theoretical atomic compositions of each polymer, initiator, and glass substrate.

**Table 2:** Characterization of mixed polymer brushes. Molecular weight, zeta potential, and contact angle.

Code	Polymer type	NIPAAm conc. (mM)	*M*_*n*_^a^	*M*_*w*_*/M*_*n*_^a^	DMAPAAm conc. (mM)	*M*_*n*_^b^	Zeta potential (mV)^c^		Contact angle (°)^d^	
							37 ​°C	20 ​°C	37°C	20 ​°C
PN	PNIPAAm	857	10,000	1.34	–	–	−0.40	−0.42	70.0 ​± ​0.0	64.0 ​± ​1.7
PN-PD-0	PNIPAAm and amino group	857	10,000	1.07	–	–	0.19	0.50	79.0 ​± ​2.6	85.7 ​± ​2.6
PN-PD-12.5	PDMAPAAm/PNIPAAm mixed brush	857	11,000	1.24	12.5	1400	4.27	−0.43	78.0 ​± ​3.6	74.3 ​± ​0.6
PN-PD-25.0	PDMAPAAm/PNIPAAm mixed brush	857	10,000	1.53	25.0	3800	7.79	0.56	78.7 ​± ​1.5	78.7 ​± ​1.7
PN-PD-37.5	PDMAPAAm/PNIPAAm mixed brush	857	10,000	1.54	37.5	15,000	9.07	1.21	77.7 ​± ​3.1	76.7 ​± ​4.9

a Determined by GPC using DMF with 50 mM LiCl as mobile phase.

b Determined by ^1^H NMR using chloroform D1.

c Determined by zeta potential analyzer.

d Determined by a contact angle meter using pure water droplet.

Surface morphology of the mixed polymer brush was obtained by AFM (Fig. 4). In PN-PD-37.5, small particles were observed, whereas similar objects were not observed in PN. The particulate matter is likely due to the aggregation of silane coupling agents through the silane coupling reaction. In the preparation of PN-PD-37.5, two types of silane coupling reagents, CPTMS and APTMS, were used. On the contrary, one type of CPTMS was used in the silane coupling reaction to prepare PN. Thus, a uniformly silane layer tends to form on the glass surface. In addition, PN-PD-37.5 exhibited relatively large surface roughness, with a root mean square (RMS) value of 3.236 ​nm. By comparison, PN exhibited a relatively small surface roughness value with an RMS of 0.946 ​nm. This result is attributed to the difference in grafted polymer structure between PN and PN-PD-37.5. PN consisted of densely packed, uniform length PNIPAAm, whereas PN-PD-37.5 consisted of the mixed structures of PNIPAAm and PDMAPAAm with different lengths of each polymer (Mn of PNIPAAm: 10,000 and Mn of PDMAPAAm:15,000, Table 2). Thus, the surface of PN-PD-37.5 exhibited a relatively rough structure compared to that of PN.

The thickness of the grafted PDMAPAAm/PNIPAAm mixed polymer brush on glass substrates was measured by ellipsometry (Fig. S6). The thickness of the PN brush was 7.92 ​± ​0.08 ​nm with relatively high accuracy (low MSE value), and the thickness was approximately the same as that in a previous report [51]. On the contrary, the thicknesses of PN-PD-0, PN-PD-12.5, PN-PD-25.0, and PN-PD-37.5 were not obtained with high accuracy (relatively high MSE values) by ellipsometry because the refractive indexes and attenuation coefficients of the mixed polymer brushes are difficult to determine. Although the obtained thickness values had low accuracy, the thicknesses of the mixed polymer brush were 12.78–18.22 ​nm, which was relatively thicker than that of PN.

Molecular weights of PNIPAAm and PDMAPAAm were obtained using GPC and ^1^H NMR, respectively (Table 2). Because the monomer concentration of NIPAAm in the ATRP was the same, PNIPAAm had nearly the same molecular weight. The molecular weight of PDMAPAAm increased as the monomer concentration increased. The results indicated that the grafted PDMAPAAm length is in the following order: PN-PD-12.5 ​< ​PN-PD-25.0 ​< ​PN-PD-37.5. This is because polymerization rate increased as the monomer concentration increased. In general polymerization, the molecular weight is determined by various parameters, such as monomer concentration, radical initiator concentration, and polymerization time. In this study, radical initiator concentration and polymerization time were the same in the RAFT polymerization. The monomer concentration of DMAPAAm was varied (12.5, 25.0, and 37.5 ​mM). Thus, the change in monomer concentration influenced the molecular weight of PDMAPAAm in the mixed brushes. The Mn of PN-PD-37.5 was 10 times larger than that of PN-PD-12.5. This result can be ascribed to the fact that RAFT polymerization was not controlled during PN-PD37.5 preparation. In the preparation condition of PN-PD-37.5, the increase in polymerization rate can be attributed to the increase in molar concentration of DMAPAAm. The excessive large polymerization rate would lose control of RAFT polymerization. Thus, PN-PD-37.5 exhibited a significantly larger molecular weight than PN-PD12.5 and PN-PD37.5.

The phase transition temperature of the mixed polymer brush cannot be directly measured because PNIPAAm was grafted to the glass substrate. However, previous reports suggested that the mixed polymer brushes exhibit a similar phase transition profile to PNIPAAm homopolymer brushes [[52], [53], [54]]. Thus, in this study, the phase transition behavior of the mixed polymer brushes would not be different from that of the PNIPAAm brush, and 37 ​°C and 20 ​°C were used as the above and below temperatures across the phase transition temperature, respectively.

The zeta potentials of the prepared mixed polymer brushes were measured (Table 2). Because of the properties of the basal glass plate, the zeta potential of the PNIPAAm homopolymer brush PN was slightly negative. PN-PD-0 exhibited a slightly positive zeta potential compared with PN, which can be attributed to the amino propyl group of APTMS. The zeta potential of the mixed polymer brush increased with increasing PDMAPAAm length: PN-PD-12.5 ​< ​PN-PD-25.0 ​< ​PN-PD-37.5. This is because the cationic property of PDMAPAAm increased as the molecular weight increased, which consequently increased the cationic property of the mixed polymer brushes. The zeta potential of the mixed polymer brushes was higher at 37 ​°C than at 20 ​°C. This result can be ascribed to the temperature-modulated exposure and concealment of PDMAPAAm caused by the shrinking and extension of PNIPAAm at 37 ​°C and 20 ​°C, respectively. At 37 ​°C, PNIPAAm in the mixed polymer brush was dehydrated and shrank, exposing the PDMAPAAm and increasing its cationic property (Fig. 1B). When the temperature was reduced to 20 ​°C, PNIPAAm was rehydrated and extended, leading to the concealed PDMAPAAm and decreased surface cationic property.

The surface hydrophobicity of the prepared mixed polymer brushes was observed by contact angle measurement (Table 2). Although the contact angle change was small, the hydrophobicity of PN was relatively larger at 37 ​°C than at 20 ​°C. This result can be attributed to the hydrophobic and hydrophilic change attributed to the dehydration and hydration of grafted PNIPAAm. However, the obvious change in contact angle with changing temperature was not observed on the mixed polymer brush, PN-PD-12.5, PN-PD-25.0, or PN-PD-37.5. This is probably due to offsetting the increased surface hydrophobicity and ionic property with increasing temperature. At 37 ​°C, the grafted PNIPAAm was dehydrated, which increased hydrophobicity. In parallel, PNIPAAm shrunk and PDMAPAAm was exposed at 37 ​°C, which increased ionic property and hydrophilicity. Thus, offset of these factors led to the unchanged contact angle value of the mixed polymer brushes with changing temperature. In addition, the mixed polymer brushes exhibited relatively larger contact angles than PN. This result may be ascribed to the increased hydrophobicity caused by the surface roughness. Surface hydrophobicity determined by contact angle increases with surface roughness [55,56]. In addition, the contact angle of the PNIPAAm-modified surface increased with increasing surface roughness [41]. Thus, relatively rough surface of the mixed polymer brushes increased hydrophobicity.

Protein adsorption is a valuable proxy to assess the cell adhesion potential of material interfaces [[57], [58], [59]]. The prepared mixed polymer brushes were immersed in fluorescent-labelled albumin and fibronectin solution, the adsorbed proteins were observed under a fluorescent microscope, and the luminance ratio of the adsorbed proteins was observed (Fig. S7). Results of the albumin adsorption measurement showed that the mixed polymer brushes PN-PD-12.5, PN-PD-25.0, and PN-PD-37.5 and PNIPAAm with amino group PN-PD-0 exhibited approximately two times larger adsorption of albumin than PN (Fig. S7A). This result can be attributed to the electrostatic interaction between cationic PDMAPAAm and albumin. Previous reports indicated that acidic proteins can be adsorbed on the PNIPAAm brush with cationic group through electrostatic interaction [60,61]. The isoelectric point of albumin is 4.8 [62]. Thus, acidic albumin was adsorbed on the mixed polymer brushes through electrostatic interaction between albumin and cationic group. In fibronectin adsorption, relatively larger amounts of fibronectin were adsorbed on the mixed polymer brushes PN-PD-12.5, PN-PD-25.0, and PN-PD-37.5 and PNIPAAm with amino group PN-PD-0 than on PN. This result can be attributed to the electrostatic interaction between fibronectin and cationic group in the mixed polymer brushes. The isoelectric point of fibronectin is 6.1 [63]. Thus, weak acidic fibronectin was adsorbed on the mixed polymer brushes through electrostatic interaction.

The zeta potentials of the mixed polymer brushes before and after incubation in DMEM with 10% FBS were observed to investigate the effect of protein adsorption on their electrostatic properties (Fig. S8). PN-PD-37.5 exhibited a large zeta potential change after incubation with cell culture medium because the proteins in the culture medium adsorbed with the cationic group of the mixed polymer brushes, which reduced the cationic properties and altered the electrostatic properties of the mixed polymer brushes. By contrast, the zeta potential of PN did not change before and after incubation with cell culture medium possibly because the protein adsorption on PN was relatively lower than that on PN-PD-37.5 (Fig. S7).

These characterizations demonstrated that the mixed polymer brushes with various PDMAPAAm lengths were prepared by changing the DMAPAAm concentration in RAFT polymerization. The mixed polymer brushes with different lengths of PDMAPAAm exhibited different cationic and protein adsorption properties. In addition, the mixed brush alter the surface cationic property of the mixed brushes was altered when the temperature was changed because of the thermoresponsive PNIPAAm shrinking.

### 3.2 Temperature-controlled selective cell adhesion and detachment

Temperature-controlled cell adhesion and detachment of the prepared mixed polymer brushes were investigated using nine types of cells. Additionally, the zeta potentials of the cells we investigated (Table S3). MSCs, BMMSCs, and HeLa cells exhibited relatively low zeta potentials. Previous reports indicated that MSCs have a relatively larger amount of sialic acid than other types of cells, such as NHDF, causing their low zeta potential [43,64]. Similarly, MSCs, BMMSCs, and HeLa cells exhibited relatively low zeta potentials.

The temperature-dependent cell adhesion and detachment of MSCs, NHDF, and HeLa cells were assessed to investigate the efficiency of MSC separation from other contaminant cells. MSCs were designated as the target cells, and NHDF and HeLa cells were used as contaminant cells. During the collection of MSCs from various tissues, MSCs are naturally mixed with other types of cells, such as fibroblast or cancer cells. Thus, NHDF and HeLa cells were used to model cell heterogeneity

**Fig. 5:**
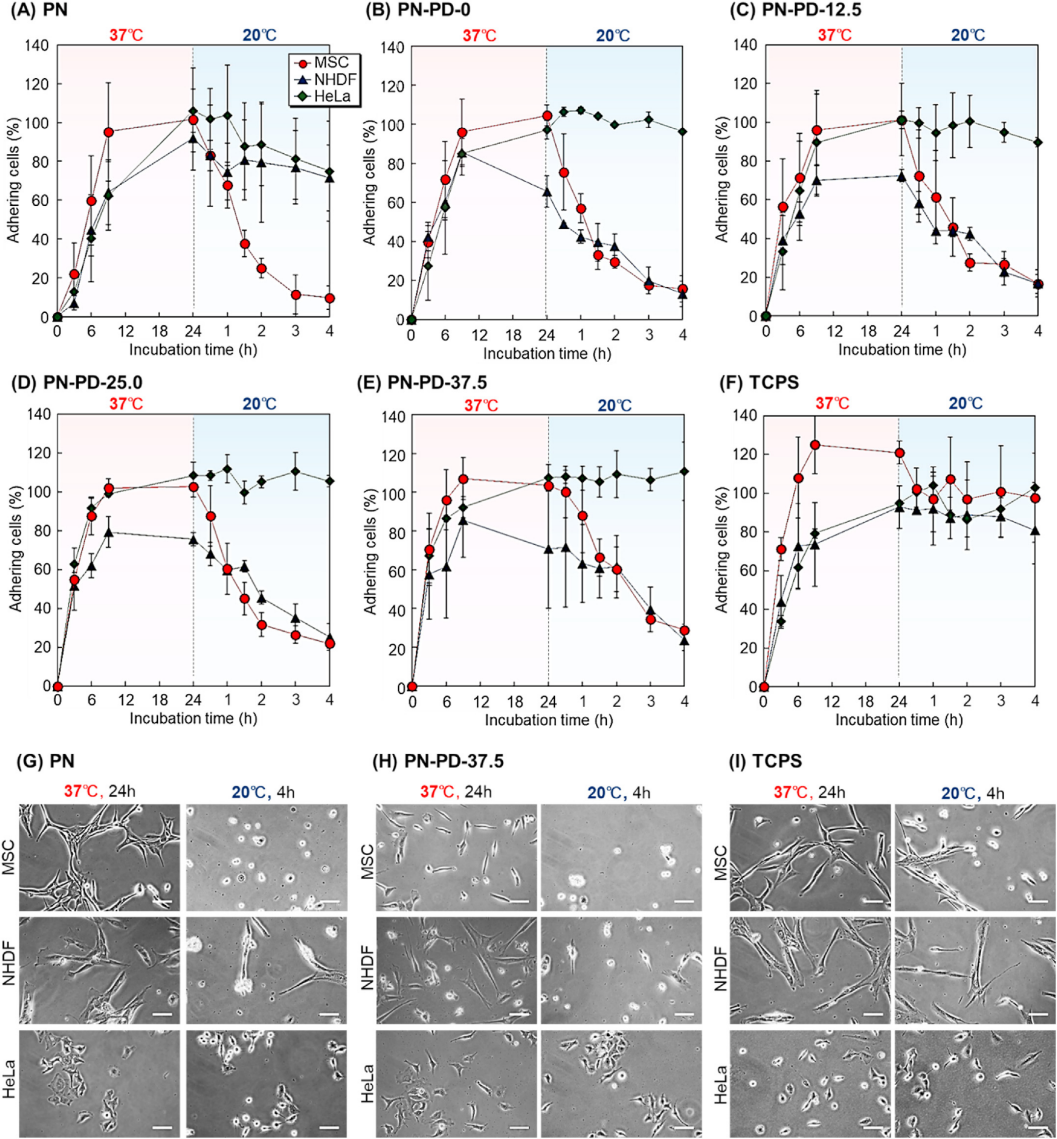
Cell adhesion and detachment profiles on mixed polymer brushes. **(A)** PN **(B)** PN-PD-0, **(C)** PN-PD-12.5, **(D)** PN-PD-25.0, **(E)** PN-PD-37.5, and **(F)** TCPS with incubation at 37 ​°C for 24 ​h and subsequent incubation at 20 ​°C for 4 ​h. Cell morphology on the prepared mixed polymer brushes **(G)** PN, **(H)** PN-PD-37.5, and **(I)** TCPS. Scale bars: 100 ​μm.

The temperature responsive cell adhesion properties of the prepared polymer brushes was investigated using MSCs, NHDF, and HeLa cells (Fig. 5A–E). As a control, cell adhesion behavior on tissue culture polystyrene (TCPS) was investigated (Fig. 5F). All cell types adhered to TCPS after 24 ​h incubation at 37 ​°C, and cells remained adhered to TCPS after 4 ​h incubation at 20 ​°C (Fig. 5F). This result is due to the fact that changing the temperature of a standard cell culture dish has no effect on the surface property. Furthermore, the MSCs, NHDF, and HeLa cells did not detach from TCPS when the temperature was lowered from 37 ​°C to 20 ​°C.

After 24 ​h incubation at 37 ​°C on PN, the majority of the seeded MSCs, NHDF, and HeLa cells adhered (Fig. 5A). Most of the adhered MSCs detached from the substrate after lowering the temperature to 20 ​°C and incubating for 4 ​h. This result can be ascribed to the thermoresponsive property of the PNIPAAm brush. At 37 ​°C, PNIPAAm was dehydrated and shrunk, which caused the cells to adhere. Meanwhile, at 20 ​°C, PNIPAAm was hydrated and extended, which caused the cells to detach. Only a small part of NHDF and HeLa detached from PN possibly because of differences in cell detachment properties between cells. Previous reports indicated that the cell detachment property of the PNIPAAm-grafted substrate influences the intrinsic cell adhesion property of the cell [65]. Thus, MSCs, NHDF, and HeLa cells exhibited different cell detachment behavior at 20 ​°C.

After 24 ​h incubation at 37 ​°C on PN-PD-0, PNIPAAm, and amino group modified substrates, the majority of MSCs and HeLa cells adhered to the brush (Fig. 5B). However, NHDF detached during the incubation at 37 ​°C possibly because of the amino group on the graft interface providing surface hydrophilicity. Thus, even at high temperatures, the slightly hydrophilic property of PN-PD-0 induced NHDF detachment. When the temperature was reduced to 20 ​°C, the adhered MSCs and NHDF detached, whereas HeLa cells still remained adhered on PN-PD-0. This result can be ascribed to the strong adhesive property on PN-PD-0 and negative zeta potential of HeLa cells (Table S3). Thus, the amino propyl group on the PN-PD-0 and HeLa cells interacted electrostatically, which caused the strong adhesion at the interface during incubation at 20 ​°C.

After 9 ​h incubation at 37 ​°C on PN-PD-12.5, PN-PD-25.0, and PN-PD-37.5, the majority of MSCs adhered (Fig. 5C, D, and 5E). MSCs exhibited relatively rapid adhesion when compared with PN possibly because the grafted PDMAPAAm on the mixed polymer brush provides cationic property to the copolymer brush. PNIPAAm with a cationic group-grafted interface enhances cell adhesion through electrostatic and hydrophobic interactions [66]. Similarly, the PNIPAAm and PDMAPAAm mixed polymer brushes enhanced cell adhesion at 37 ​°C, which consequently shortened the required adhesion time. The MSC adhesion rate increased as the PDMAPAAm length was increased possibly because the surface cationic property increased as the PDMAPAAm length was increased, which led to cell adhesion induced by the strong electrostatic interactions between cells and PDMAPAAm.

The adhesion rates of MSCs and NHDF slightly decreased from 9 to 24 ​h on PN-PD-25.0 and PN-PD-37.5 possibly because of the strong cationic property of the mixed polymer brush providing the hydrophilic property, which caused the slight detachment of the cells during incubation. MSCs and NHDF cells detached from PN-PD-12.5, PN-PD-25.0, and PN-PD-37.5 when the temperature was reduced to 20 ​°C, whereas HeLa cells remained adherent. This result can be ascribed to the difference in adhesion and detachment behavior among the cells.

The cell morphologies of the PN, PN-PD-37.5, and TCPS after 24 ​h incubation at 37 ​°C and 4 ​h incubation at 20 ​°C were compared (Fig. 5G, H, and 5I). MSCs and NHDF cells spread relatively slowly on PN-PD-37.5 compared with those on PN and TCPS, indicating that the cationic property suppressed their spreading behavior. By contrast, HeLa cells spread more on PN-PD-37.5 than on PN and TCPS possibly because of the cationic property of PN-PD-37.5.

These results indicated that the mixed polymer brushes PN-PD-12.5, PN-PD-25.0, and PN-PD-37.5 enhanced cell adhesion at 37 ​°C, which can attributed to the electrostatic and hydrophobic interactions. Additionally, HeLa cells still adhered on the brushes after incubation at 20 ​°C.

**Fig. 6:**
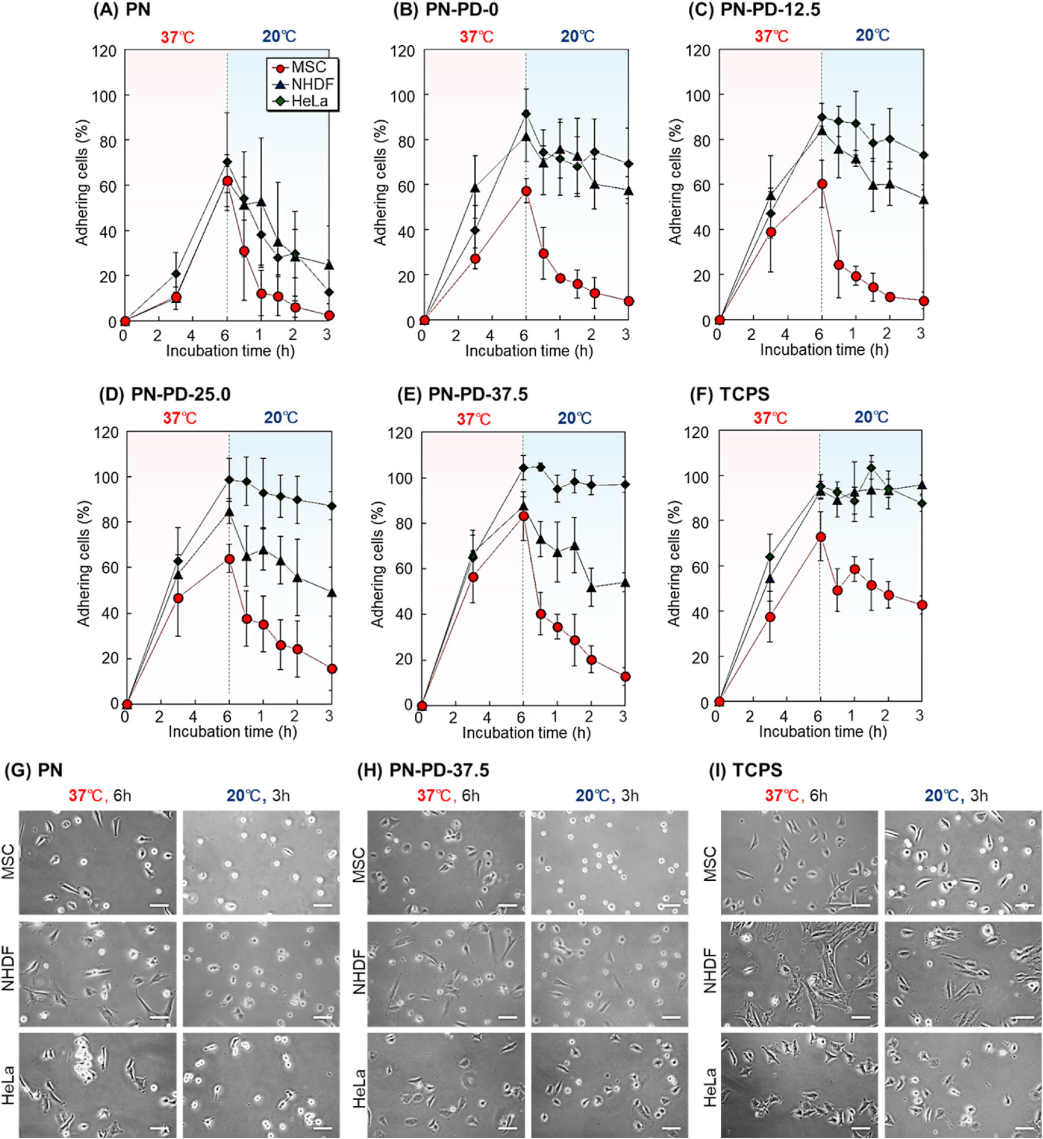
Cell adhesion and detachment profiles on mixed polymer brushes. **(A)** PN, **(B)** PN-PD-0, **(C)** PN-PD-12.5, **(D)** PN-PD-25.0, **(E)** PN-PD-37.5, and **(F)** TCPS with incubation at 37 ​°C for 6 ​h followed by incubation at 20 ​°C for 3 ​h. Cell morphology on the prepared mixed polymer brushes **(G)** PN, **(H)** PN-PD-37.5, and **(I)** TCPS. Scale bars: 100 ​μm.

The mixed polymer brushes exhibited rapid adhesion of MSCs. Thus, short incubation times at 37 ​°C for 6 ​h and 20 ​°C for 3 ​h were investigated (Fig. 6). After 6 ​h incubation at 37 ​°C, the majority of MSCs adhered to the brush on PN-PD-37.5, whereas other polymer brushes had a relatively low MSC adhesion ratio. This result indicated that the mixed polymer brush with long PDMAPAAm can shorten the incubation time at 37 ​°C (Fig. 6E). Additionally, a different cell detachment behavior from the mixed polymer brushes was observed on PN-PD-37.5. MSCs promptly detached, and NHDF gradually detached during incubation at 20 ​°C, whereas HeLa cells remained adhered during incubation at 20 ​°C. Previous report indicated that two types of cells, vascular endothelial cells and myoblast cells, are separated using different cell detachment rates of PNIPAAm brush [65]. Similarly, PN-PD-35 exhibited different cell detachment rates during incubation at 20 ​°C. Thus, the prepared mixed polymer brush PN-PD-37.5 should be utilized for temperature-modulated cell separation at different cell detachment rates. By contrast, all cells showed a similar detachment behavior on PN during incubation at 20 ​°C (Fig. 6A). The results indicated that cationic PDMAPAAm can influence the detachment behavior of the cells.

The morphologies of the cells on the prepared PN, PN-PD-37.5, and TCPS were compared after 6 ​h incubation at 37 ​°C and 3 ​h incubation at 20 ​°C. (Fig. 6G, H, and 6I). Relatively small spreading of MSC, NHDF, and HeLa cells was observed on PN, PN-PD-37.5, and TCPS at 37 ​°C incubation for 6 ​h compared to the incubation at 37 ​°C for 24 ​h, because the cell spreading was sufficiently conducted during a 6-h incubation at 37 ​°C versus a 24-h incubation. Despite the fact that complete cell spreading was not achieved after 6 ​h incubation, cells adhered to the polymer brush. There was a difference in cell detachment behavior between 6 and 24 ​h incubation (Fig. 5, Fig. 6), which is probably attributed to the difference in cell spreading on polymer brushes. Large spread cells on polymer brush took a longer time to detach than the small spread cells, resulting in a difference in cell detachment behavior after 24 ​h and 6 ​h incubation at 37 ​°C.

**Fig. 7:**
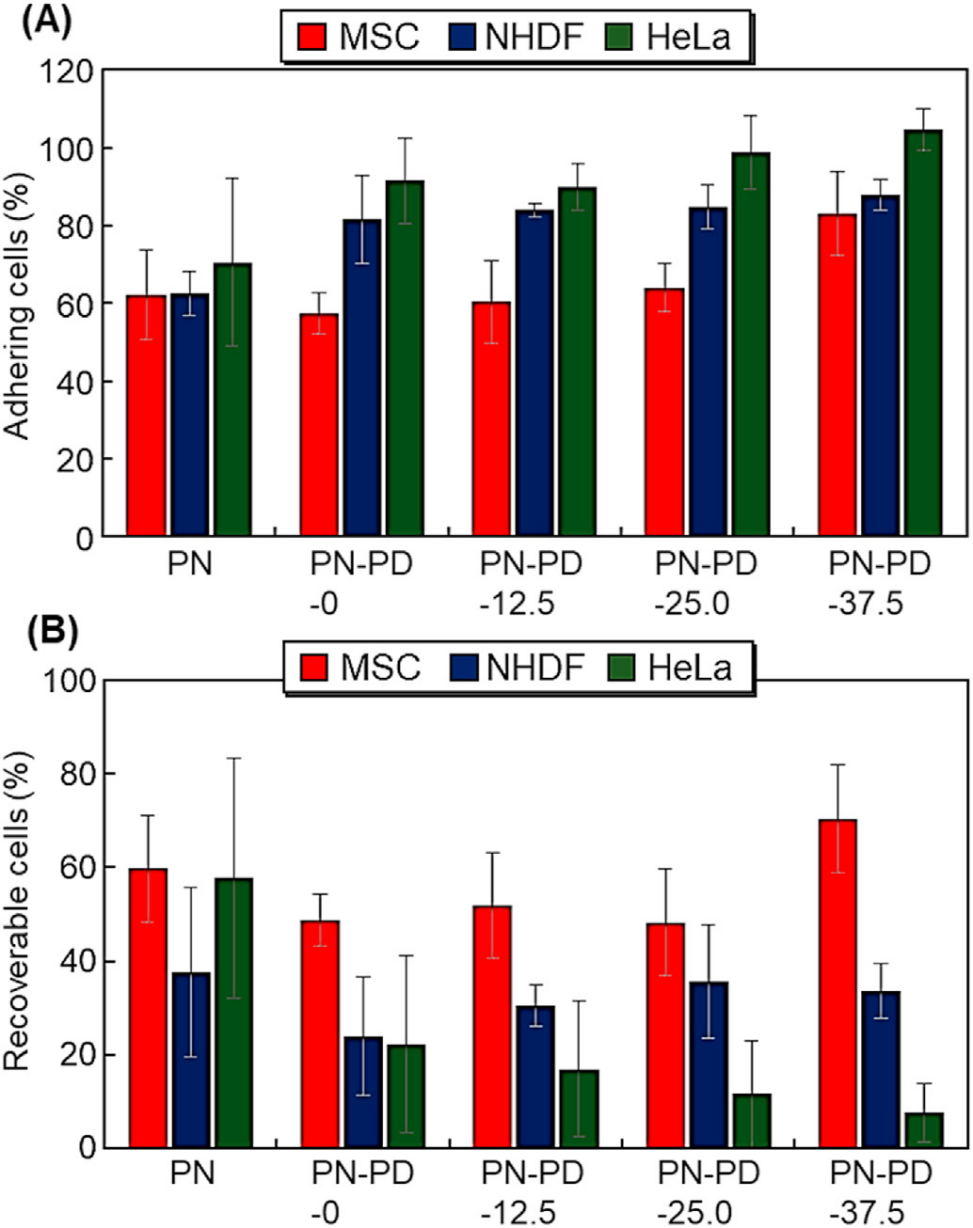
(A) Cell adhesion ratio after incubation at 37 ​°C for 6 ​h. **(B)** Recovered cells after incubation at 37 ​°C for 6 ​h followed by incubation at 20 ​°C for 3 ​h.

The adhered cell ratio after incubation at 37 ​°C for 6 ​h was summarized (Fig. 7A). The adhesion of most cells increased as the cationic property of the polymer brush increased as such, PN ​< ​PN-PD-0 ​< ​PN-PD-12.5 ​< ​PN-PD-25.0 ​< ​PN-PD-37.5. The results indicated that cell adhesion can be enhanced by incorporating cationic property in the brush. The cells were recovered after incubation at 37 ​°C for 6 ​h followed by incubation at 20 ​°C for 3 ​h (Fig. 7B). MSC recovery was relatively high in PN-PD-37.5, whereas NHDF and HeLa recovery was quite low. High MSC recovery and low contaminant cell recovery are desired in MSC separation. Thus, PN-PD-37.5 would be suitable for temperature-modulated MSC separation.

**Fig. 8:**
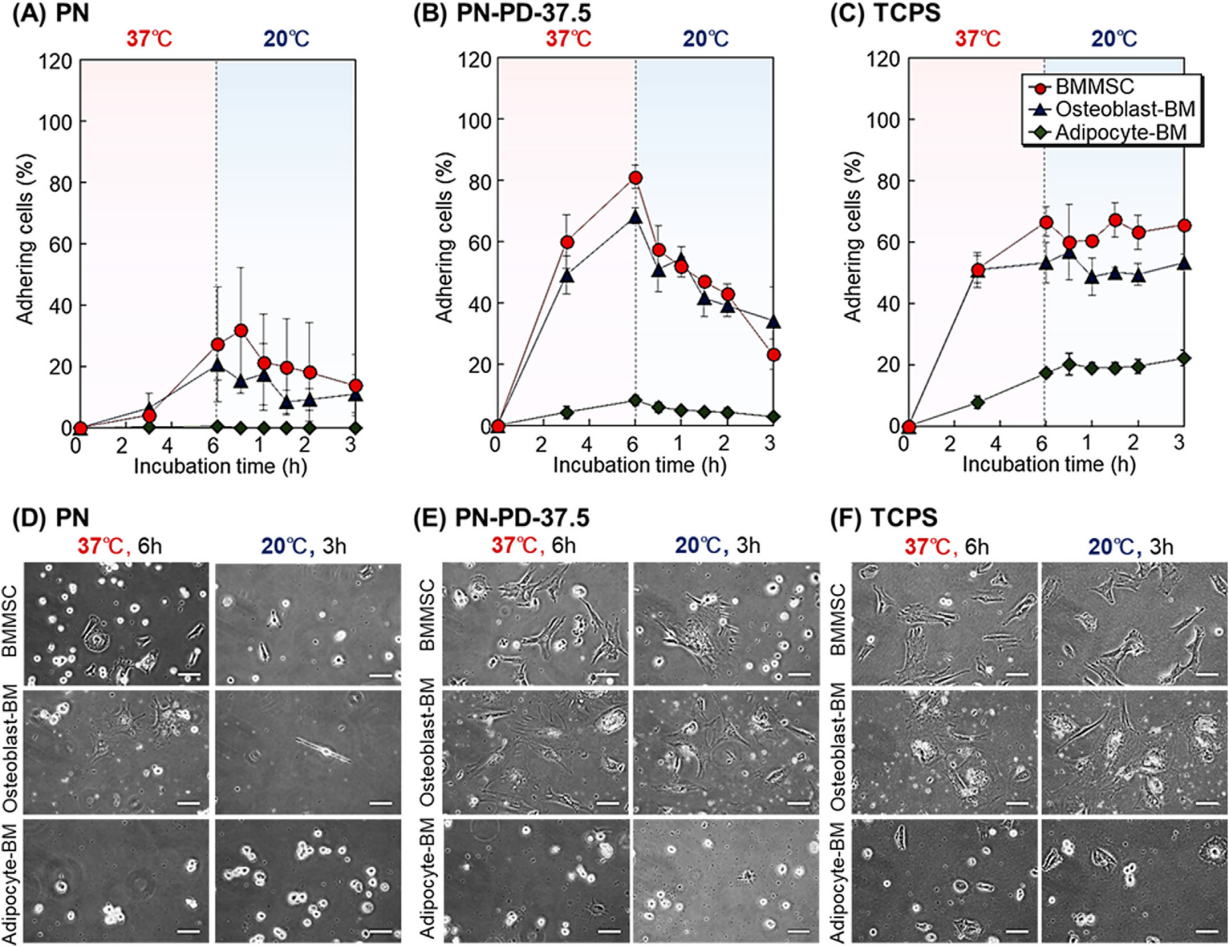
Cell adhesion and detachment profiles of BMMSC, osteoblast-BM, and adipocyte-BM on **(A)** PN, **(B)** PN-PD-37.5, and **(C)** TCPS with incubation at 37 ​°C for 6 ​h followed by incubation at 20 ​°C for 3 ​h. Cell morphology on **(D)** PN, **(E)** PN-PD-37.5, and **(F)** TCPS. Scale bars: 100 ​μm.

The prepared mixed polymer brush PN-PD-37.5 was then investigated as a cell separation material for the primary culture of MSCs and their osteogenic and adipogenic differentiated cells. Human BMMSCs were osteogenically and adipogenically differentiated into osteoblasts (osteoblast-BM) and adipocytes (adipocyte-BM), respectively (Fig. S1). The temperature-dependent cell adhesion and detachment profiles of these cells were observed (Fig. 8). BMMSCs and osteoblast-BM adhered to TCPS at 37 ​°C, but the adipocytes did not (Fig. 8C). The result indicated that BMMSCs and osteoblast-BM adhered on the cell culture plate, while adipocytes did not. On PN, three types of cells did not adhere after incubation at 37 ​°C (Fig. 8A). This result indicated that the hydrophobicity of PNIPAAm brush was not suitable for the adhesion of these cells. PN-PD-37.5, on the other hand, demonstrated BMMSC and osteoblast-BM adhesion after 37 ​°C incubation (Fig. 8B). Thus, the introduction of PDMAPAAm into the polymer brush enhanced the adhesion of BMMSCs and osteoblast-BM. Adherent BMMSCs and osteoblast-BM detached from the mixed polymer brush when the temperature was reduced to 20 ​°C. Adipocyte-BM did not adhere on the mixed polymer brush because of the low adhesion property of adipocytes.

Furthermore, we compared the cell morphology on the prepared PN, PN-PD-37.5, and TCPS after incubation at 37 ​°C for 6 ​h followed by incubation at 20 ​°C for 3 ​h (Fig. 8D, E, and 8F). Similar spreading of BMMSCs was observed on PN-PD-37.5 and TCPS. On the other hand, BMMSC spreading on PN was not observed. This result indicated that introducing PDMPAAm in PN-PD-37.5 contributed to the spreading of cells.

**Fig. 9:**
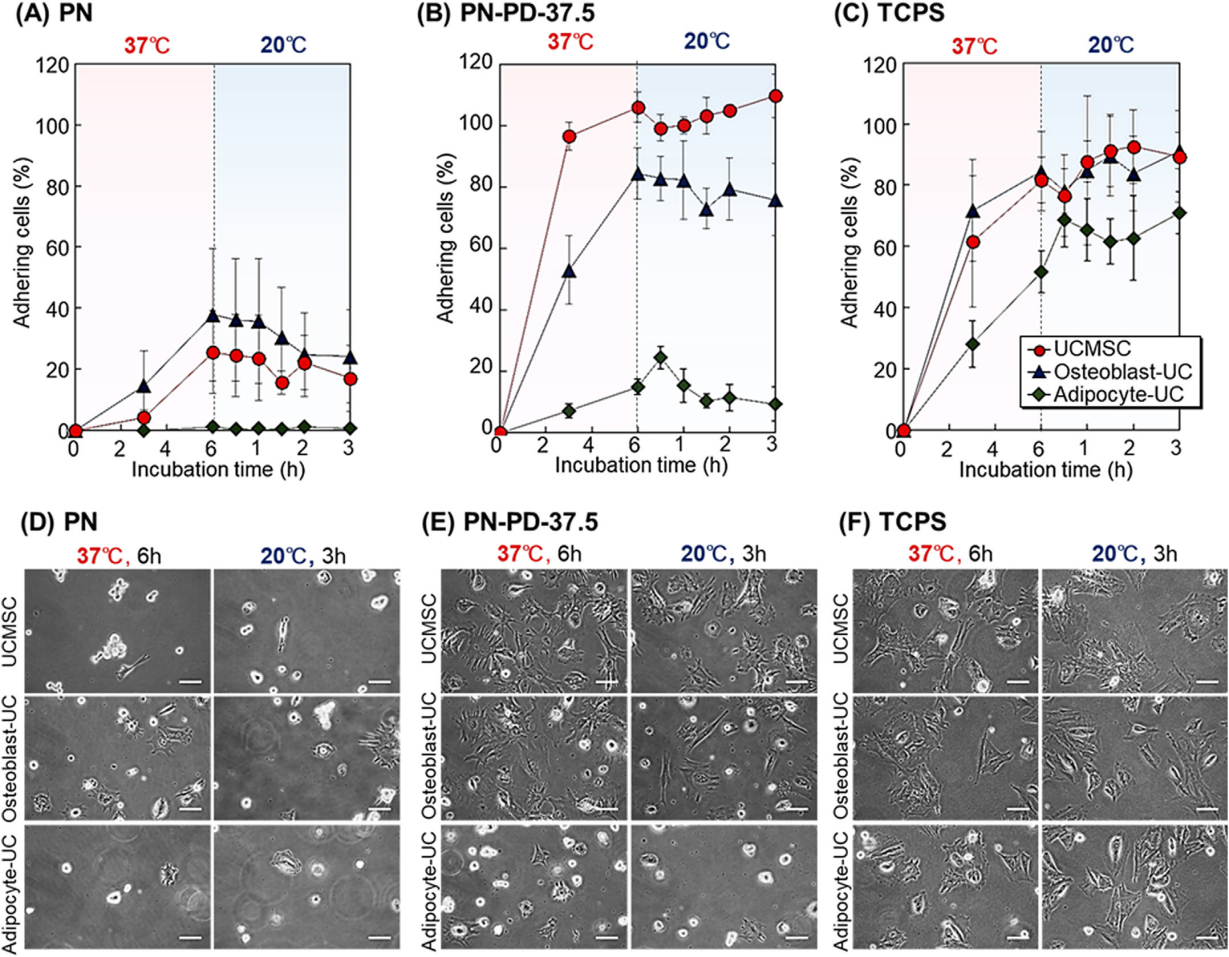
Cell adhesion and detachment profiles of UCMSC, osteoblast-UC, and adipocyte-UC on **(A)** PN, **(B)** PN-PD-37.5, and **(C)** TCPS with incubation at 37 ​°C for 6 ​h followed by incubation at 20 ​°C for 3 ​h. Cell morphology on **(D)** PN, **(E)** PN-PD-37.5, and **(F)** TCPS. Scale bars: 100 ​μm.

The adhesion and detachment properties of the mixed polymer brushes of umbilical cord-derived MSCs (UCMSCs) and their osteogenic and adipogenic differentiated cells were investigated (Fig. 9). UCMSCs were osteogenically and adipogenically differentiated into osteoblasts (osteoblast-UC) and adipocytes (adipocyte-UC), respectively (Fig. S1).

After 6 ​h incubation at 37 ​°C, UCMSC and osteoblast-UC adhered to TCPS (Fig. 9C). The adhesion of adipocyte-UC on TCPS was relatively lower than those of UCMSCs and osteoblast-UC because of the low adhesion property of adipocytes. However, low adhesion of UCMSC, osteoblast-UC, and adipocyte-UC was observed after incubation at 37 ​°C (Fig. 9A) because the hydrophobicity of PNIPAAm at 37 ​°C was not suitable for cell adhesion. By contrast, the mixed polymer brushes exhibited high UCMSC and osteoblast-UC adhesion at 37 ​°C (Fig. 9B) because the introduced cationic property of the mixed polymer brush enhanced the cell adhesion compared with the non-cationic PNIPAAm brush. Adipocyte-UC did not adhere to the mixed polymer brushes at 37 ​°C because of the low adhesive property of adipocytes. These results indicated that PN-PD-37.5 exhibited a different cell adhesion property, making it a suitable cell separation material.

**Fig. 10:**
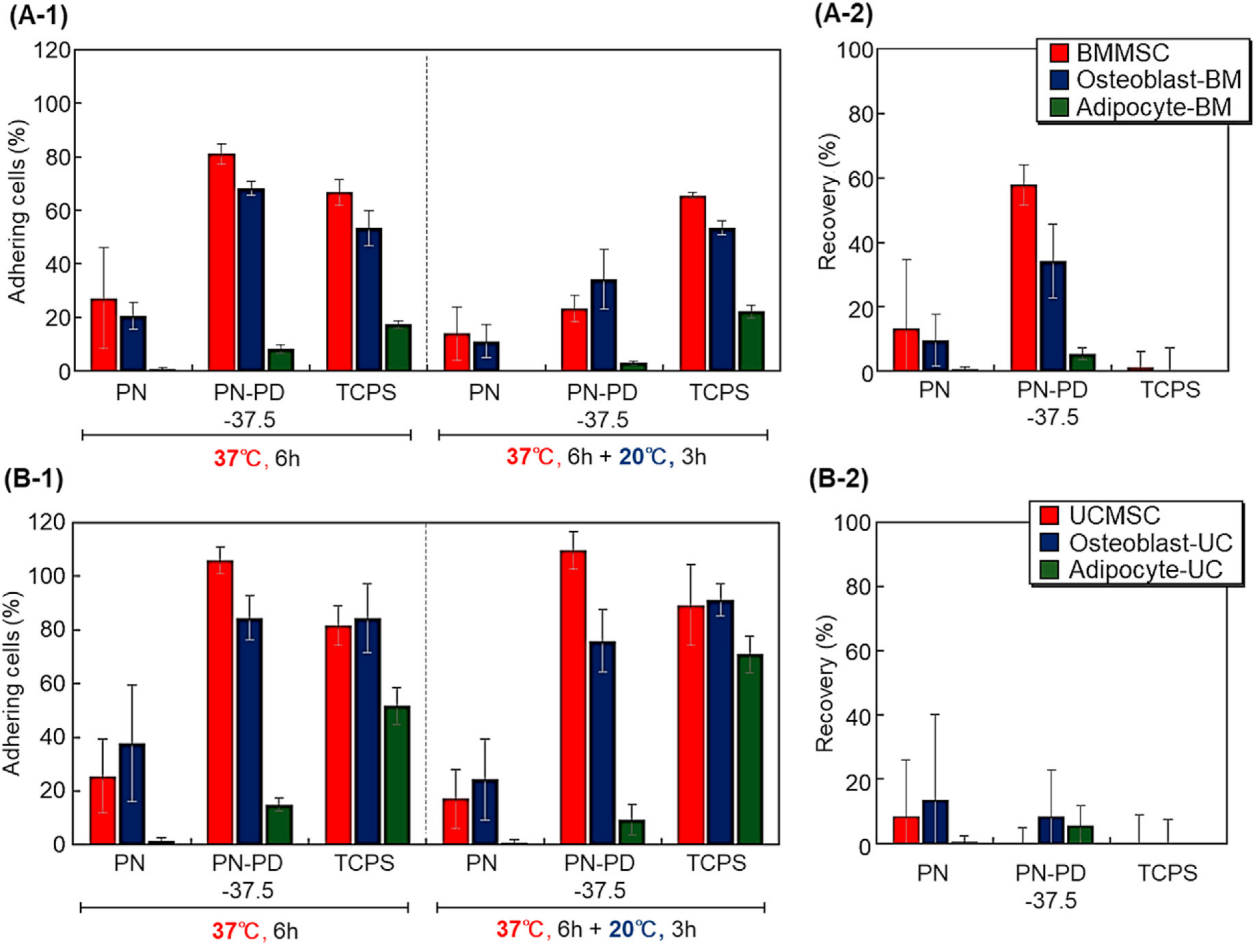
(A-1) Cell adhesion ratio of BMMSC, osteoblast-BM, and adipocyte-BM after incubation at 37 ​°C for 6 ​h and subsequent incubation at 20 ​°C for 3 ​h. (A-2) Recovery ratio of BMMSC, osteoblast-BM, and adipocyte-BM. (B-1) Cell adhesion ratio of UCMSC, osteoblast-UC, and adipocyte-UC after incubation at 37 ​°C for 6 ​h followed by incubation at 20 ​°C for 3 ​h. (B-2) Recovery ratio of UCMSC, osteoblast-UC, and adipocyte-UC.

The adhesion properties of BMMSCs and UCMSCs and their differentiated cells on the prepared substrates were compared (Fig. 10). At 37 ​°C, PN-PD-37.5 demonstrated BMMSC and UCMSC adhesion (Fig. 10A). The results indicated that PN-PD-37.5 is a suitable substrate for the adhesion of MSCs. When the temperature was changed from 37 ​°C to 20 ​°C, the adhered BMMSCs detached from the prepared mixed polymer brush, whereas USMSCs remained adhered to the mixed polymer brush (Fig. 10B). The difference in detachment property can be attributed to the strong adhesive property of UCMSCs. UCMSCs exhibited a high adhesion ratio on PN-PD-37.5, indicating that these cells strongly adhered on the mixed polymer brush. Thus, the adhered UCMSCs did not detach from the mixed polymer brush when the temperature was reduced. The results indicated that the prepared mixed polymer brush would be suitable for the temperature-modulated adhesion and detachment of BMMSCs rather than UCMSCs.

**Fig. 11:**
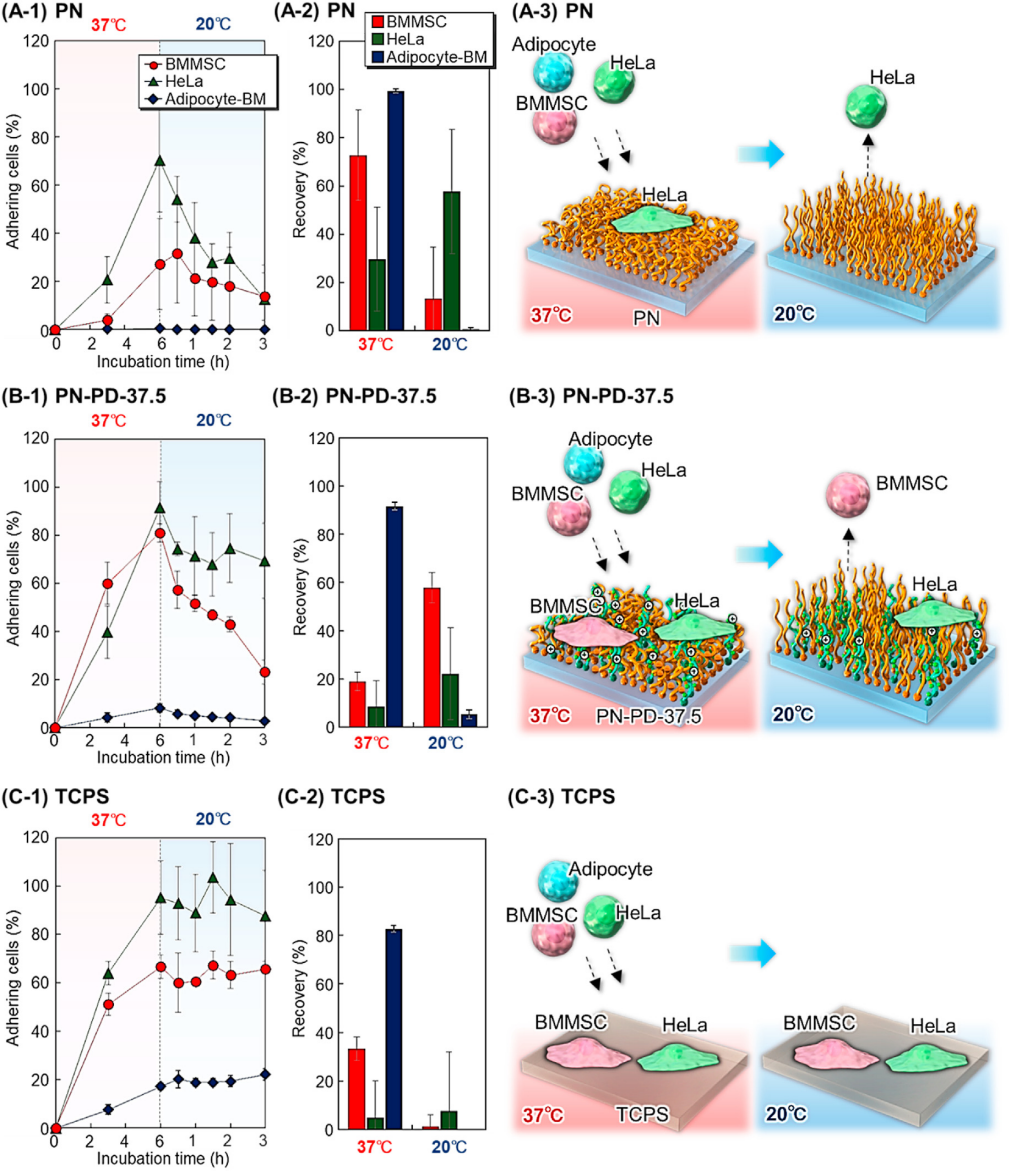
Comparison of cell adhesion behavior for estimating cell separation performance on (A) PN, (B) PN-PD-37.5, and (C) TCPS. (1) Cell adhesion ratio on each substrate, (2) recovery of cells at 37 ​°C and 20 ​°C, and (3) schematic diagram of cells separated using each substrate.

By comparing cell adhesion behavior of BMMSC, adipocyte, and HeLa cells, the prepared substrates were investigated as temperature-modulated cell separation materials. (Fig. 11). At 37 ​°C, BMMSCs and HeLa cells adhered to the TCPS, whereas adipocyte-BM did not (Fig. 11C). Thus, purified adipocyte-BM can be obtained at 37 ​°C (Figs. 11C–2). The adhered BMMSCs and adipocyte-BM remained adhered to TCPS after the temperature was reduced. Thus, to recover the adhered BMMSCs and HeLa cells, a digestive enzyme such as trypsin is needed, which would reduce the cell function [67]. These results suggest that TCPS is an unsuitable substrate for cell separation.

At 37 ​°C, HeLa cells adhered relatively well to PN, whereas BMMSCs and adipocyte-BM did not (Fig. 11A). At 37 ​°C, non-adherent BMMSCs and adipocyte-BM can be obtained (Figs. 11A–2). The adhered HeLa cells were detached from PN when the temperature was lowered to 20 ​°C, which resulted in HeLa cell recovery (Figs. 11A–2). Thus, PN can be used to separate HeLa cells from other BMMSCs and adipocyte-BM by changing the temperature.

BMMSC and HeLa cells adhered to the mixed polymer brush PN-PD-37.5 ​at 37 ​°C, whereas adipocyte-BM did not (Fig. 11B). Thus, a high composition of adipocytes can be obtained at 37 ​°C (Figs. 11B–2). The adhered BMMSCs were detached from PN-PD-37.5 when the temperature was lowered to 20 ​°C, whereas HeLa cells remained adhered (Fig. 11B–A). Thus, a large composition of BMMSCs can be obtained at 20 ​°C. These results indicated that PN-PD-37.5 can perform temperature modulation on three types of cells simply by changing the temperature. Thus, PN-PD-37.5 can be used to separate undifferentiated and differentiated stem cells or remove cancer cells from stem cells by changing the temperature.

**Fig. 12:**
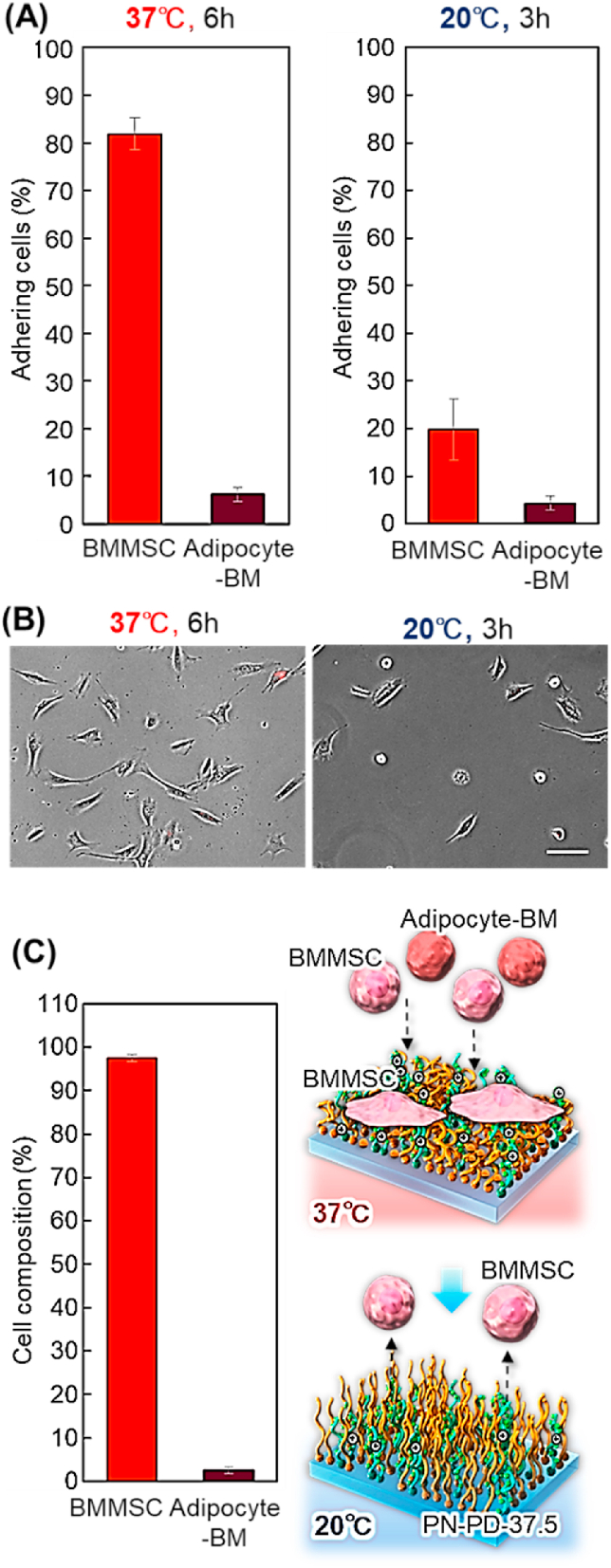
Cell separation of a mixture of BMMSCs and adipocyte-BM using the prepared PDMAPAAm/PNIPAAm mixed brush (PN-PD-37.5). **(A)** Cell adhesion with incubation at 37 ​°C for 6 ​h using DMEM with FBS (10%) and subsequent incubation at 20 ​°C for 3 ​h using DMEM with FBS (10%). Data are expressed as the mean value with standard deviation (n ​= ​3). **(B)** Cell morphology on the prepared block copolymer brushes. BMMSC: unstained. Adipocyte-BM: red. Scale bars: 100 ​μm. **(C)** Recovered cell composition after incubation at 20 ​°C. (For interpretation of the references to color in this figure legend, the reader is referred to the Web version of this article.)

Separation of the BMMSCs and adipocyte-BM mixture was performed using the prepared PN-PD-37.5 mixed brush (Fig. 12). Unstained BMMSCs and red stained adipocyte-BM (1:1) suspension was prepared and seeded to the mixed polymer brush PN-PD-37.5 ​at 37 ​°C and incubated for 6 ​h. Most of seeded BMMSCs were adhered on the mixed polymer brush PN-PD-37.5, whereas most of adipocytes were not adhered on the mixed brush (Fig. 12A and B). The result indicated that most of the adipocyte-BM were collected from culture medium at 37 ​°C. When the temperature was reduced to 20 ​°C, most of adhered BMMSCs were detached from PN-PD-37.5. Thus, BMMSCs could be recovered from the culture medium after incubation at 20 ​°C. Small amounts of BMMSCs remained on PN-PD-37.5, which was slightly different from the adhesion and detachment from only BMMSC seeding. This is probably because the secreted extracellular matrix in co-culture conditions with BMMSCs and adipocyte-BM slightly prevents the detachment of BMMSCs. The high composition of BMMSCs was obtained after incubation at 20 ​°C (Fig. 12C). These results suggest that MSCs can be separated from differentiated cells simply by changing the temperature.

In addition, the BMMSCs were separated from HeLa cells by using PN-PD-37.5 (Fig. S9). A suspension of unstained BMMSCs and green stained HeLa cells (1:1) was prepared and seeded to PN-PD-37.5 ​at 37 ​°C and then incubated for 6 ​h. BMMSCs and HeLa cells were adhered on PN-PD-37.5 (Figs. S9A and B). When the temperature was reduced to 20 ​°C, most of adhered BMMSCs were detached from PN-PD-37.5, whereas HeLa cells were not detached from PN-PD-37.5. Thus, BMMSCs could be recovered from the culture medium after incubation at 20 ​°C. Small amounts of BMMSCs remained on PN-PD-37.5, a result slightly different from the adhesion and detachment from only BMMSC seeding. This result may be ascribed to the fact that the secreted extracellular matrix in the co-culture conditions with BMMSCs and HeLa cells slightly prevented the detachment of BMMSCs. The high composition of BMMSCs was obtained after incubation at 20 ​°C (Fig. S9C). These results suggest that MSCs can be separated from contaminant cells by simply changing the temperature.

The recovered BMMSCs were osteogenically and adipogenically differentiated to investigate the undifferentiated potency of the recovered cells from the mixed polymer brush (Fig. S10). The recovered BMMSCs were positive for Alizarin Red S staining, indicating that the recovered BMMSCs retained their osteogenic differentiation ability. The recovered BMMSCs were also cultured in an adipogenic differentiation medium. The recovered BMMSCs were positive for Oil Red staining, indicating that the recovered BMMSCs retained their adipogenic differentiation ability. In our previous study, we investigated the mRNA expression of the MSCs recovered from PNIPAAm brush-grafted microfibers [40]. The recovered MSCs with adipogenic differentiation exhibit similar PPARγ and LPL expression levels to MSCs without seeding to the PNIPAAm brush and with adipogenic differentiation. We also confirmed the expression of CD90 on MSCs recovered from the PDMAPAAm-*b*-PNIPAAm brush through flow cytometry [68]. Thus, the MSCs recovered from the mixed polymer brushes would also have differentiated potency similar to previously reported cell separation materials using PNIPAAm copolymer brushes [40,68].

These results indicated that the developed mixed polymer brush can enhance cell adhesion compared with the PNIPAAm homopolymer brush and conventional cell culture plates owing to the enhanced electrostatic interaction between cells and the incorporated PDMAPAAm. The mixed polymer brush would be useful for the temperature-modulated separation of cells because of its different cell adhesion and detachment properties among various cell types. Thus, the developed mixed polymer brush would be a functional temperature responsive biomaterial for tissue engineering using MSCs.

## 4 Conclusions

We developed mixed polymer brushes composed of thermoresponsive PNIPAAm and cationic PDMAPAAm via a combination of surface-initiated RAFT polymerization and ATRP. The prepared mixed polymer brushes exhibited increased cationic property and grafted PDMAPAAm length. The surface cationic property can be controlled by changing the temperature because of the shrinking and extension of PNIPAAm, which exposed and concealed PDMAPAAm, respectively. The prepared mixed polymer brushes can enhance cell adhesion at 37 ​°C through their electrostatic interactions with cells. They also exhibited temperature-controlled adhesion and detachment properties on various types of cells. BMMSCs adhered on the mixed polymer brushes at 37 ​°C and detached at 20 ​°C, whereas HeLa cells adhered on the mixed polymer brushes at 37 ​°C and did not detach at 20 ​°C. Adipocytes did not adhere on the mixed polymer brushes at 37 ​°C. Using the prepared mixed polymer brush, we separated MSCs from adipocytes and HeLa cells by simply changing the temperature. Thus, the developed mixed polymer brushes could be used as functional biomaterials for the separation of MSCs from their differentiated or contaminant cells by altering temperature.

## Credit author statement

**Kenichi Nagase**: Conceptualization, Writing - Review & Editing, Supervision, **Haruno Wakayama**: Methodology, Investigation, **Junnosuke Matsuda**: Methodology, Investigation, **Naoto Kojima**: Methodology, Investigation, **Hideko Kanazawa**: Supervision.

## Funding

This research was supported by a Grant-in-Aid for Scientific Research (grant nos. 19H02447, 20H05233, 22H04560, 21KK0199, and 22K19899) from the 10.13039/501100001691Japan Society for the Promotion of Science, Japan. This work was partly supported by the 10.13039/100015280Precise Measurement Technology Promotion Foundation (PMTP-F), and 10.13039/501100008656Iketani Science and Technology Foundation.

## Declaration of competing interest

The authors declare that they have no known competing financial interests or personal relationships that could have appeared to influence the work reported in this paper.

## Data Availability

Data will be made available on request.
