# Supplementary material for: Thermoresponsive mixed polymer brush to effectively control the adhesion and separation of stem cells by altering temperature
Source: Mater Today Bio. 2023 Apr 7;20:100627. doi: 10.1016/j.mtbio.2023.100627 (PMC10130502; doi:10.1016/j.mtbio.2023.100627)
Supplement: Multimedia component 1 [file mmc1.docx]

*Supplementary Materials*

Thermoresponsive Mixed Polymer Brush to Effectively Control the Adhesion and Separation of Stem Cells by Altering Temperature

Kenichi Nagase*, Haruno Wakayama, Junnosuke Matsuda, Naoto Kojima, Hideko Kanazawa

Faculty of Pharmacy, Keio University, 1-5-30 Shibakoen, Minato, Tokyo 105-8512, Japan

*Corresponding author Tel: +81-3-5400-1378; Fax: +81-3-5400-1378

E-mail: nagase-kn@pha.keio.ac.jp, nagase.kenichi@keio.jp

**S.1 Materials**

*N*-isopropylacrylamide (NIPAAm) and *N,N*-dimethylamino propylacrylamide (DMAPAAm) were obtained from KJ Chemicals (Tokyo, Japan). *N,N*-dimethylformamide (DMF), methanol, acetone, tris(2-aminoethyl) amine, ascorbic acid, toluene, 4,4'-Azobis(4-cyanovaleric acid) (V-501), 2-propanol, tris(2-aminoethyl)amine (TREN), copper(II) chloride (CuCl_2_), ethylenediamine-*N,N,N',N'*-tetraacetic acid (EDTA), and 1,4-dioxane were purchased from Fujifilm (Osaka, Japan). Tris[(2-dimethylamino)ethyl]amine (Me_6_TREN) was prepared from TREN. *α*-Chloro-*p*-xylene and 3-aminopropyl trimethoxysilane (APTMS) were purchased from Tokyo Chemical Industry (Tokyo, Japan). (Chloromethyl)phenylethyl-trimethoxysilane (CPTMS) was obtained from Gelest (Morrisville, PA, USA). 1-Ethoxycarbonyl-2-ethoxy-1,2-dihydroquinoline (EEDQ) and 2-(dodecylthiocarbonothioylthio)-2-methylpropionic acid were purchased from Sigma–Aldrich (St Louis, MO, USA). Glass substrate (24 mm × 50 mm, thickness: 0.17–0.25 mm) was purchased from Matsunami Glass Industry (Osaka, Japan). Bone marrow-derived mesenchymal stem cells (BMMSCs) were obtained from JCRB Cell Bank (Osaka, Japan). Normal human dermal fibroblasts (NHDF) and HeLa cells were obtained from Lonza (Basel, Switzerland) and RIKEN BRC (Tsukuba, Japan), respectively. Human BMMSCs, human umbilical cord matrix-derived MSCs (UCMSCs), and osteogenic and adipogenic differentiation media were purchased from Promocell GmbH (Heidelberg, Germany). These cells were cultured using cell culture medium with supplements (Table S1). Dulbecco’s modified Eagle’s medium (DMEM) (low glucose, pyruvate), DMEM (high glucose), and minimum essential media (MEM) were purchased from Thermo Fisher Scientific (Waltham, MA, USA).

**S.2 Cell culture**

Human BMMSCs, NHDF, and HeLa cells were cultured as described below using cell culture media (Table S1). Cells were cultured on a 100 mm tissue culture polystyrene dish (TCPS) at 37 °C in 5% CO_2_ using a CO_2_ incubator (9000EX, Wakenbtech, Kyoto, Japan). The cell culture medium was changed every 3 days. At 80% confluency, the medium was removed by aspiration. Then, the cells were rinsed with PBS (2 mL), and trypsin solution (2.5 mL) was added to the dish. The dish was incubated for 3 min. After the detachment of the cells from the dish, the cell culture medium (2.5 mL) was added to the dish, and the cell suspension was collected to a centrifuge tube. Centrifugation was performed at 1500 rpm for 3 min. The supernatant was removed, and the medium was added to the cells. A 10 μL aliquot of the cell suspension was collected, and the cells in the suspension were counted. The cells were seeded to 3.5×10^3^ cells/cm^2^ to the 100 mm TCPS for the passage culture.

**Table S1** Cell culture media

| Cell | Culture media ^a)^ | Additives ^b)^ |
| --- | --- | --- |
| Human bone marrow mesenchymal stem cells: MSCs | DMEM, low glucose, pyruvate | FBS (10%)  bFGF (3 ng/mL)  Penicillin–streptomycin (1%) |
| Normal human dermal fibroblasts: NHDF | DMEM, high glucose, pyruvate | FBS (10%)  NEAA (1%)  Penicillin–streptomycin (1%) |
| HeLa cells | MEM | FBS (10%)  NEAA (1%)  Penicillin–streptomycin (1%) |

a) Volume of cell culture medium was 500 mL. b) Additives were added to 500 mL of cell culture medium.

Human BMMSCs and human UCMSCs were cultured as described below using cell culture media (Table S2). Cells were cultured on a 100 mm TCPS dish at 37 °C in 5% CO_2_ using a CO_2_ incubator (9000EX, Wakenbtech, Kyoto, Japan). The cell culture medium was changed every 3 days. At 80% confluency, the medium was removed by aspiration. Then, the cells were rinsed with PBS (2 mL), and TrypLE Express (2.5 mL) was added to the dish. The dish was incubated for 3 min. After the detachment of the cells from the dish, the cell culture medium (2.5 mL) was added to the dish, and the cell suspension was collected to a centrifuge tube. Centrifugation was performed at 1200 rpm for 3 min. The supernatant was removed, and the medium was added to the cells. A 10 μL aliquot of the cell suspension was collected, and the cells in the suspension were counted. The cells were seeded to 6.0×10^3^ cells/cm^2^ to the 100 mm TCPS for the passage culture.

**Table S2** Cell culture media

| Cell | Culture media ^a)^ | Additives ^b)^ |
| --- | --- | --- |
| Human bone marrow derived mesenchymal stem cells (BMMSCs) | DMEM, low glucose,  no _L_-glutamine, no phenol red | FBS (10％)  NEAA (1％)  Penicillin–streptomycin (1%)  Gluta Max (1%) |
| Human umbilical cord matrix derived mesenchymal stem cells (UCMSCs) | DMEM, low glucose, no _L_-glutamine, no phenol red | FBS (10％)  NEAA (1％)  Penicillin–streptomycin (1%)  Gluta Max (1%) |

a) Volume of cell culture medium was 500 mL. b) Additives were added to 500 mL cell culture medium.


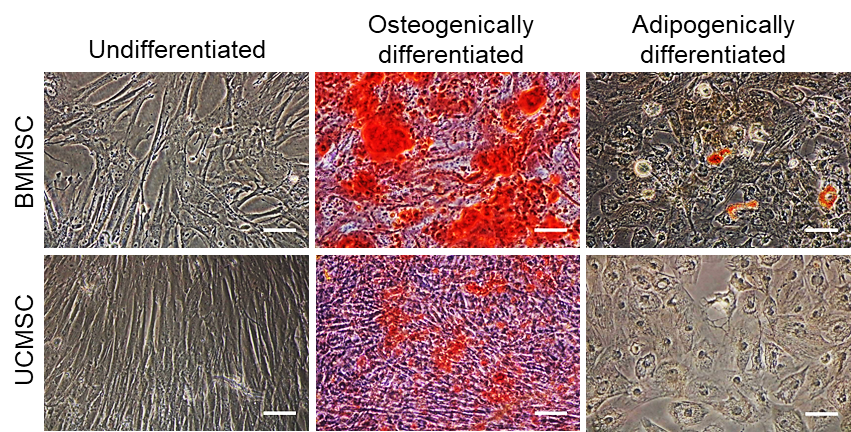
Osteogneic and adipogenic differentiation of BMMSCs and UCMSCs were performed as following. BMMSCs and UCMSCs were seeded on a 35 mm TCPS dish at 1.0×10^5^ cells/dish in 5% CO_2_ using a CO_2_ incubator (9000EX, Wakenbtech, Kyoto, Japan) for 3 days until confluence was reached. Then, the culture media were removed by aspiration, and osteogenic and adipogenic differentiation media were added. Cells were cultured for 16 days with the medium changed every 3 days. Osteogenic and adipogenic differentiation was confirmed by staining with Alizarin Red S and Sudan III, respectively.

**Fig. S1** Confirmation of osteogenically and adipogenically differentiation of BMMSCs and UCMSCs.

**
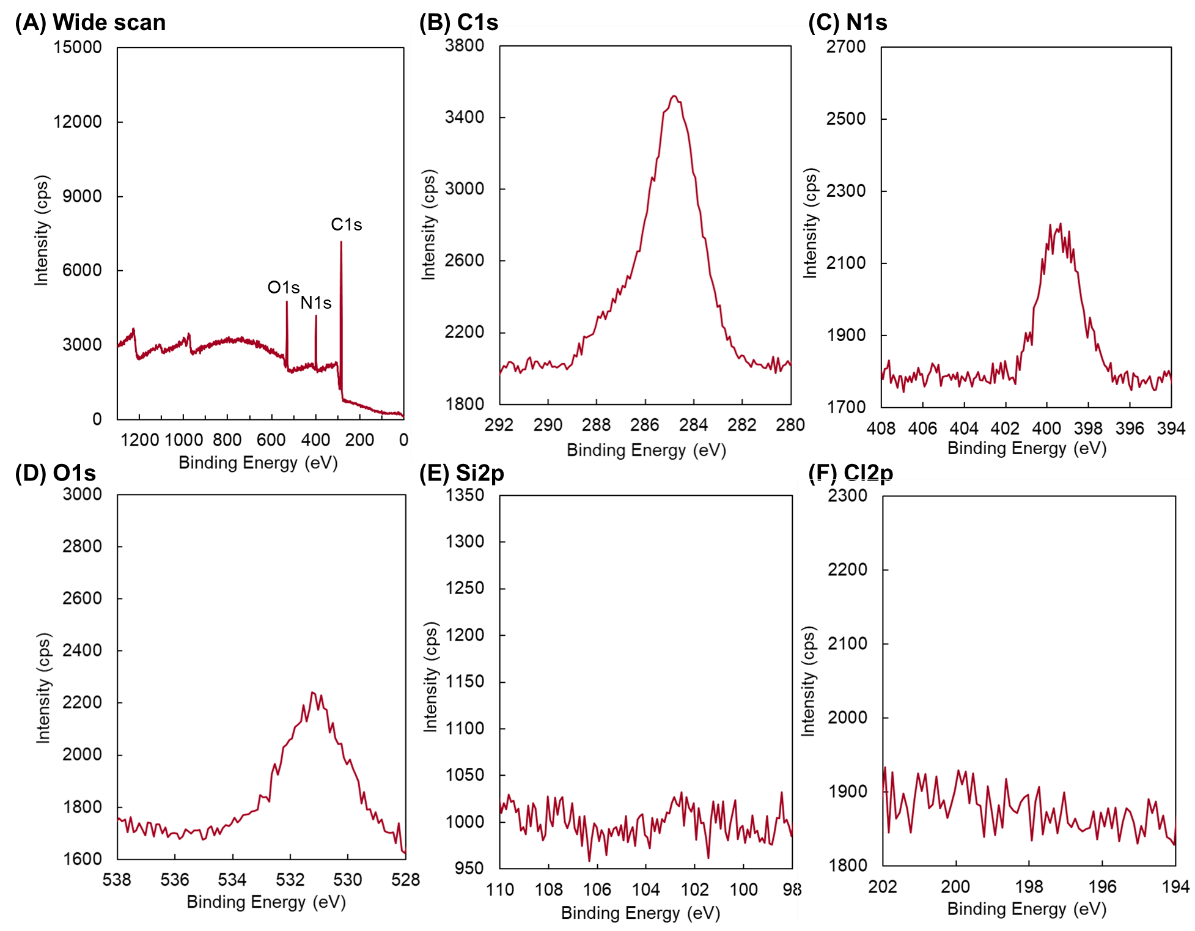
**

**Fig. S2** XPS spectrum of PNIPAAm brush-grafted glass substrate (PN). Take-off angle of 15°.

**
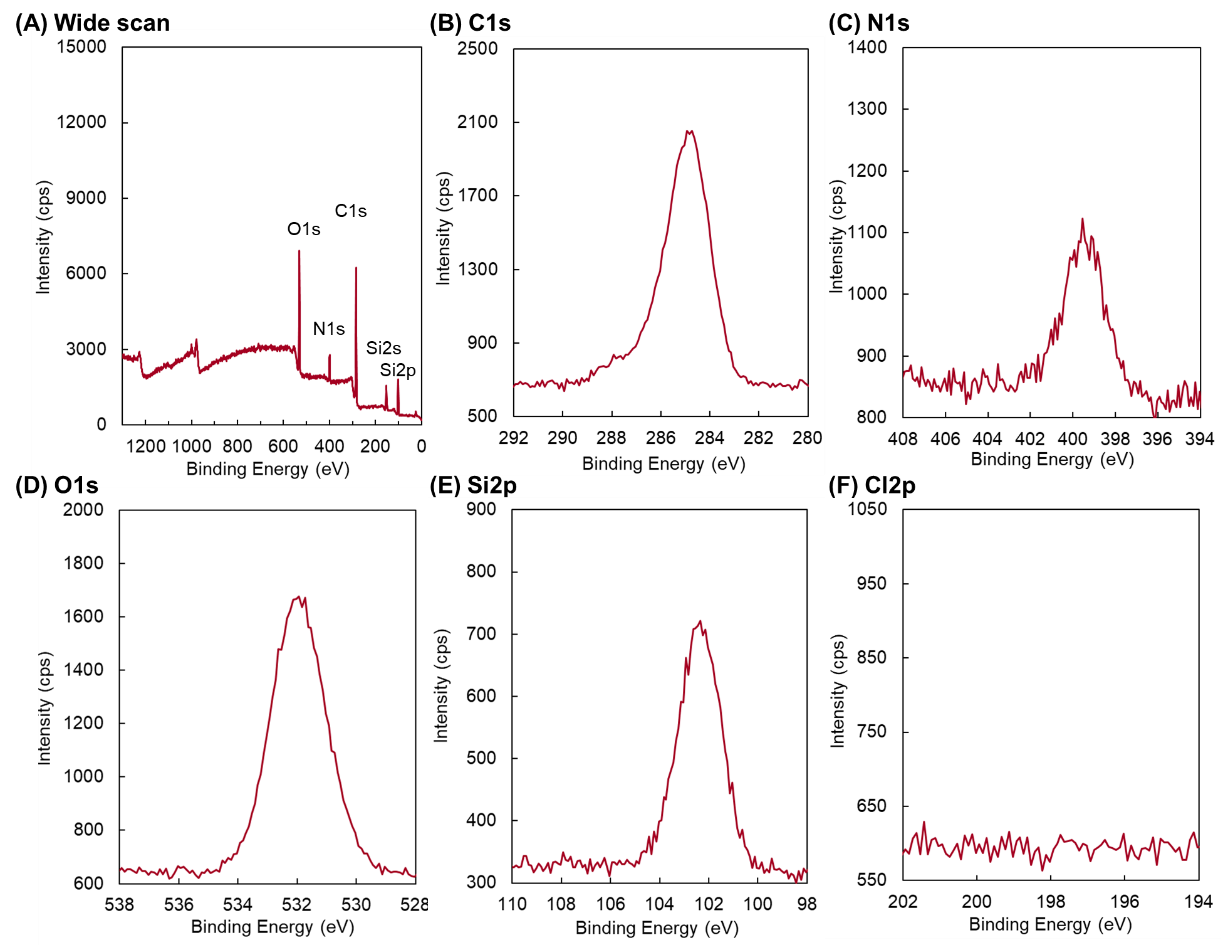
**

**Fig. S3** XPS spectrum of PNIPAAm brush-grafted glass substrate (PN-PD-0). Take-off angle of 15°.

**
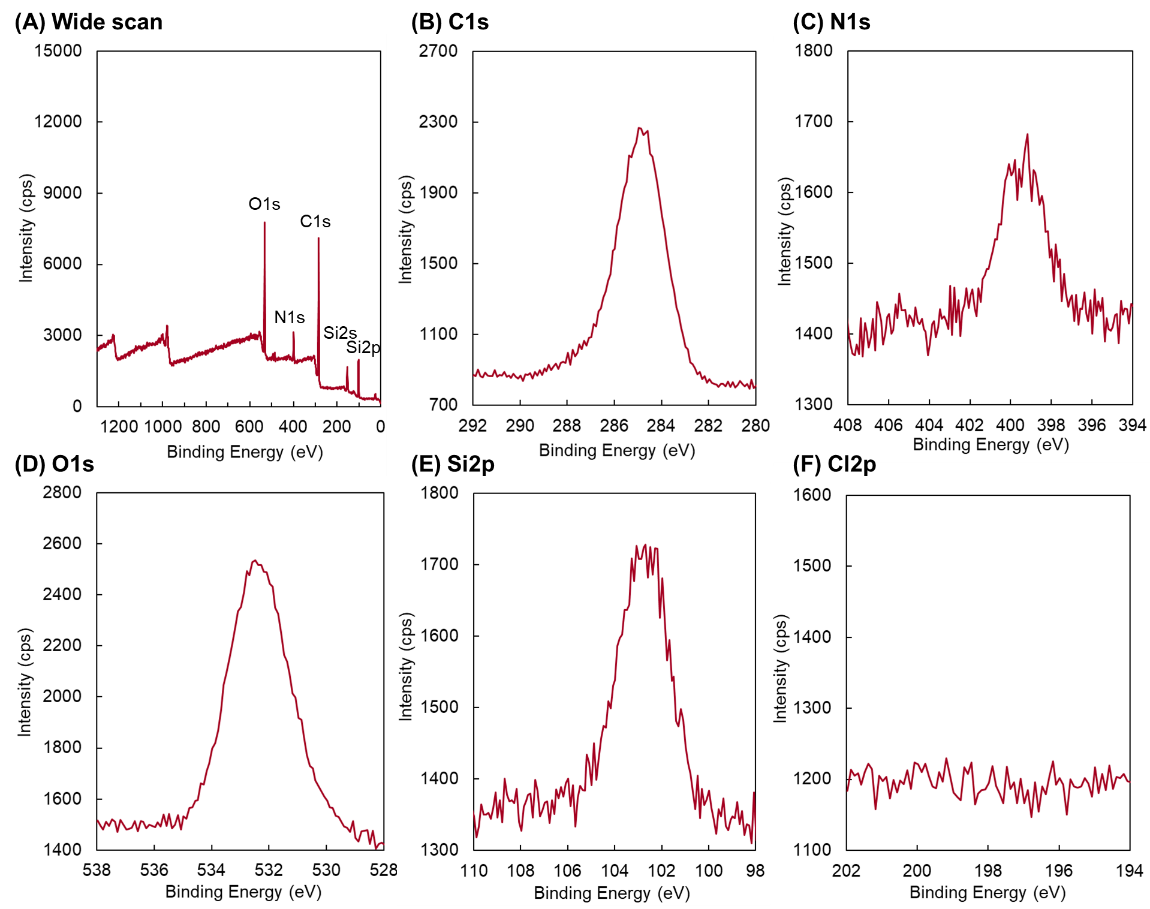
**

**Fig. S4** XPS spectrum of PDMAPAAm/PNIPAAm mixed brush-grafted glass substrate (PN-PD-12.5). Take-off angle of 15°.

**
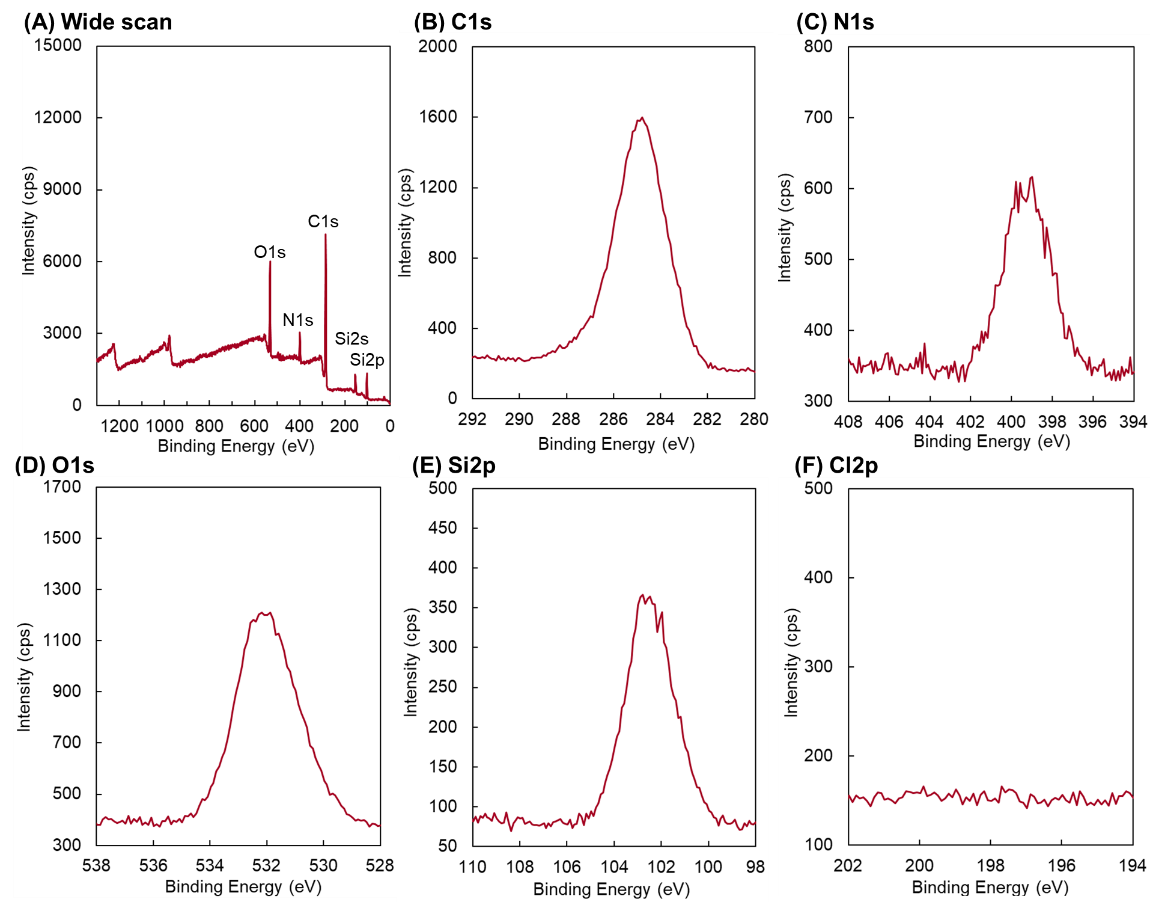
**

**Fig. S5** XPS spectrum of PDMAPAAm/PNIPAAm mixed brush-grafted glass substrate (PN-PD-25.0). Take-off angle of 15°.

**
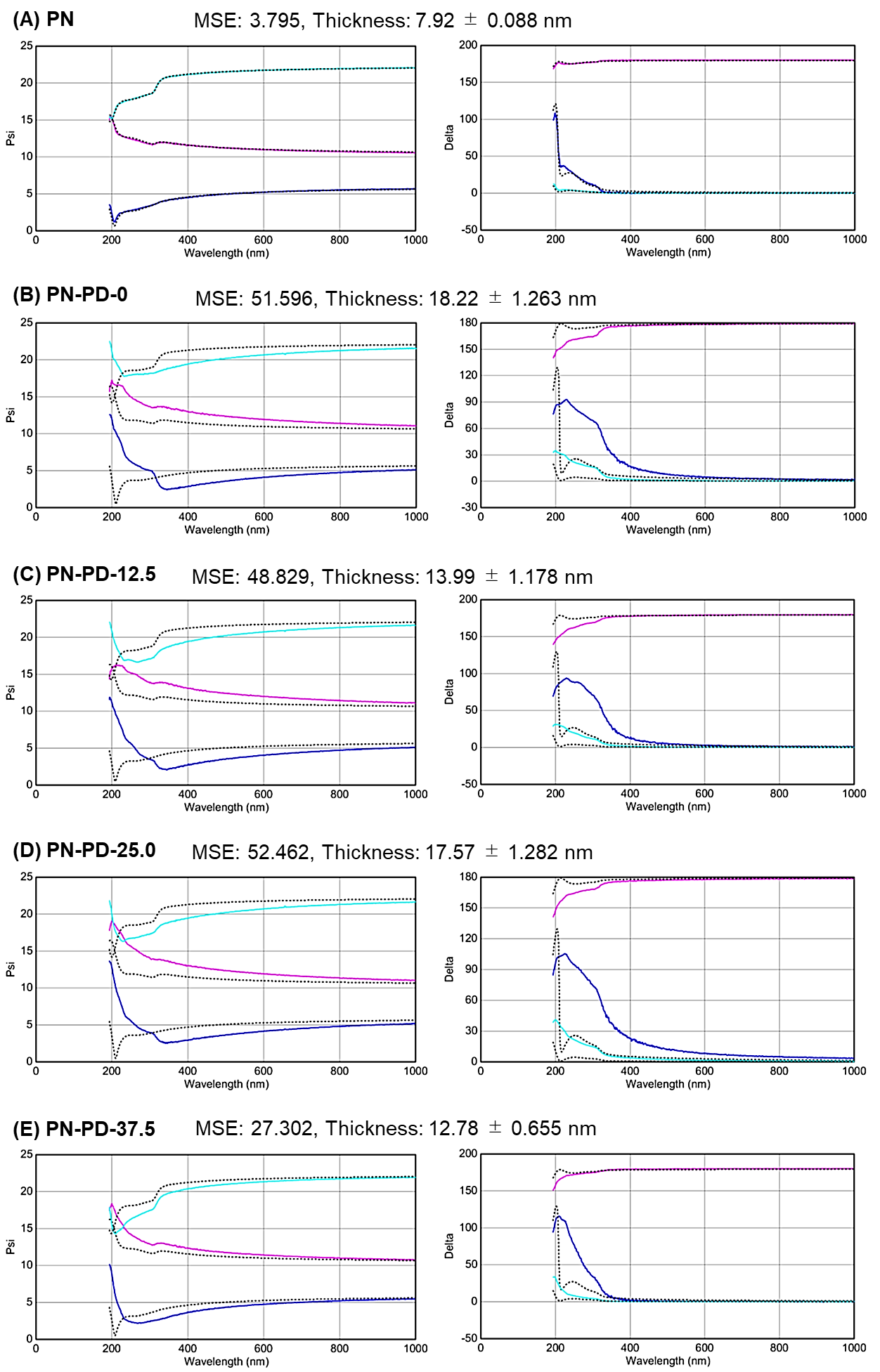
**

**Fig. S6** *Ψ* and *Δ* spectra obtained ellipsometry measurement of the prepared mixed polymer brush. **(A)** PN, **(B)** PN-PD-0, **(C)** PN-PD-12.5, **(D)** PN-PD-25.0, and **(E)** PN-PD-37.5. Pink, blue, and watery lines are measurement values obtained with incident angles of 50°, 60°, and 70°. Dotted lines are calculated values using the optical model.

**
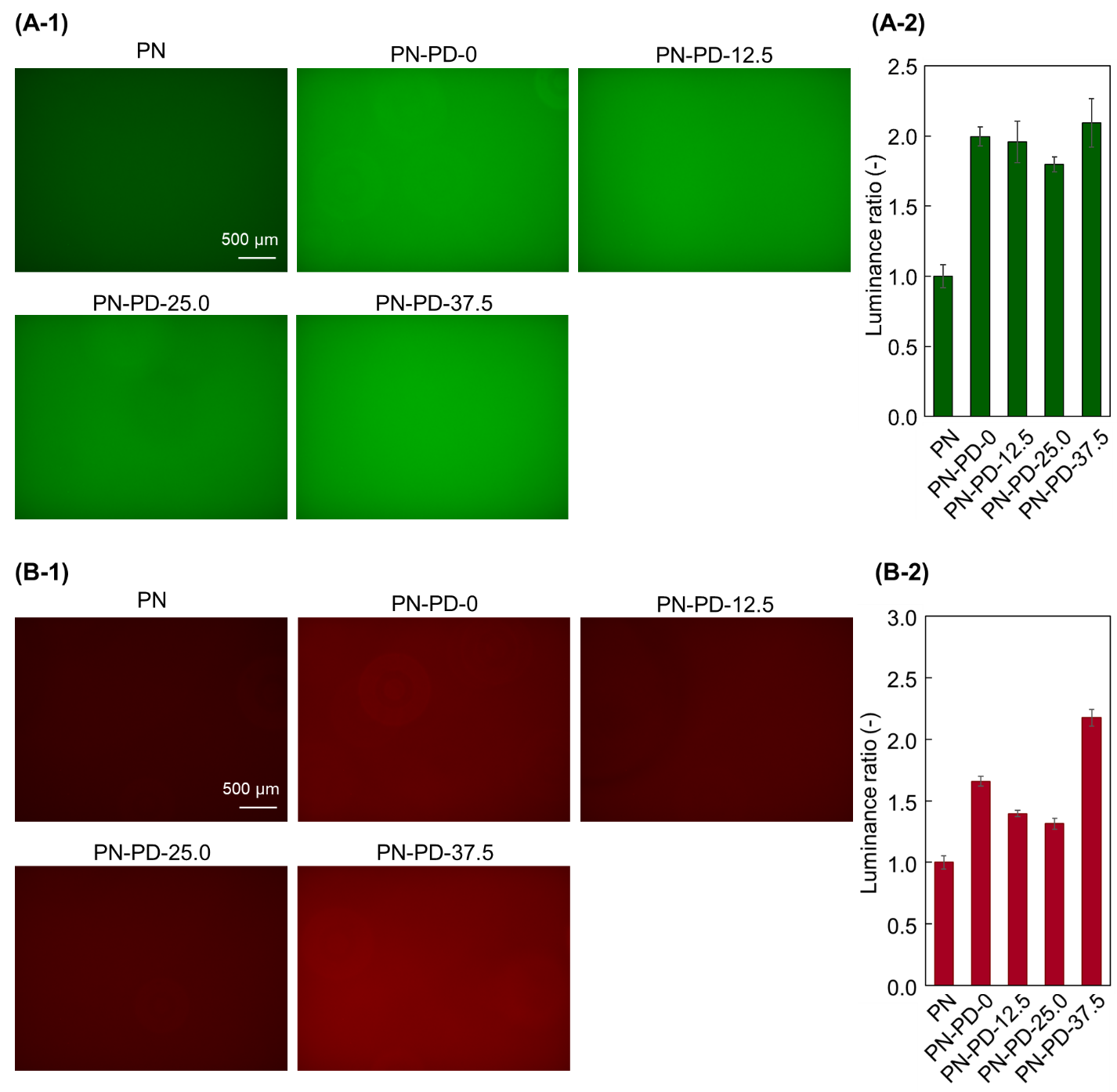
**

**Fig. S7** Protein adsorption of the prepared PDMAPAAm/PNIPAAm mixed brush-grafted glass substrates. (A-1) Fluorescence images of the adsorbed fluorescein isothiocyanate (FITC)-conjugated albumin on the prepared mixed brushes. (A-2) Luminance ratio of the FITC albumin-adsorbed mixed polymer brushes. (B-1) Fluorescence images of the adsorbed rhodamine-conjugated fibronectin on the prepared mixed brushes. (B-2) Luminance ratio of the rhodamine-conjugated fibronectin-adsorbed mixed polymer brushes.

**
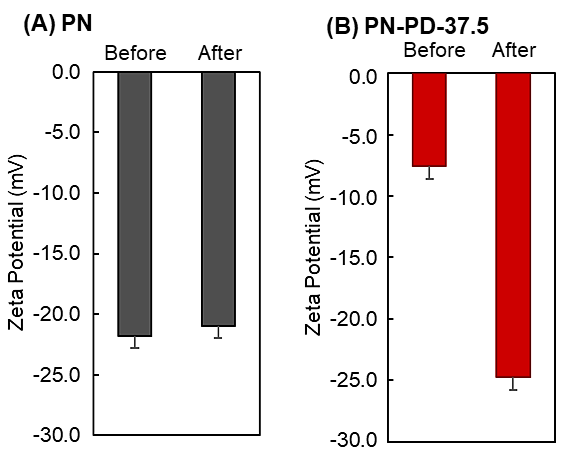
**

**Fig. S8** Zeta potential of the mixed polymer brush before and after incubation with cell culture medium.

**S.3 Zeta potential measurement**

The zeta potentials of the cells were measured to investigate their electrostatic properties. An aqueous solution of sodium dihydrogen phosphate (31.2 g/L, 19 mL) and sodium hydrogen phosphate solution (28.4 g/L, 81 mL) was mixed. The pH of the mixed solution was confirmed to be 7.4. The solution was diluted to 200 mL. Subsequently, 1 mL of the diluted solution and 9 mL of PBS were mixed. Sucrose (0.85 g) was dissolved in this solution. The cells were suspended in the prepared solution at a density of 5.0 × 10^5^ cells/mL. The cell suspension was placed in a zeta potential analyzer (ELSZ2KOP; Otsuka Electronics, Osaka, Japan). The zeta potentials of the cells were measured at 37 °C.

**Table S3.** Zeta potential of the cells.

| Cell | Zeta potential (mV) ^a)^ |
| --- | --- |
| MSC | −22.37 ± 8.86 |
| NHDF | −5.39 ± 1.77 |
| HeLa | −10.48 ± 0.51 |
| BMMSC | −18.76 ± 2.82 |
| Osteoblast-BM | −6.24 ± 0.60 |
| Adipocyte-BM | −10.77 ± 0.33 |
| UCMSC | −6.26 ± 0.92 |
| Osteoblast-UC | −12.12 ± 1.15 |
| Adipocyte-UC | −12.99 ± 1.86 |

1. Measured with a zeta potential analyzer.


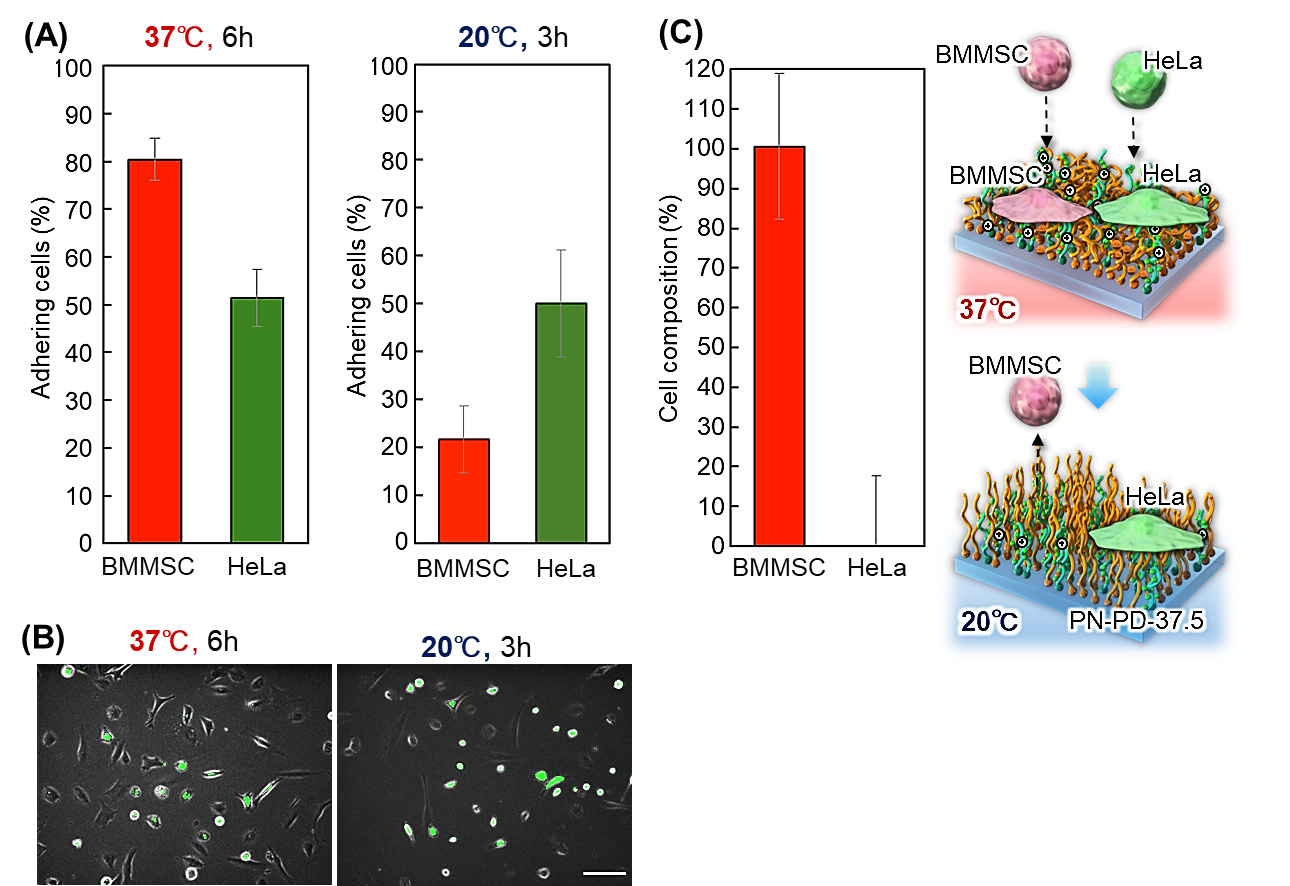


**Fig. S9** Cell separation of a mixture of BMMSCs and HeLa cells using the prepared PDMAPAAm/PNIPAAm mixed brush (PN-PD-37.5). (A) Cell adhesion with incubation at 37 °C for 6 h using DMEM with FBS (10%) and subsequent incubation at 20 °C for 3 h using DMEM with FBS (10%). Data are expressed as the mean value with standard deviation (n = 3). (B) Cell morphology on the prepared block copolymer brushes. BMMSC: unstained. HeLa cells: green. Scale bars: 100 μm. (C) Recovered cell composition after incubation at 20 °C.

**
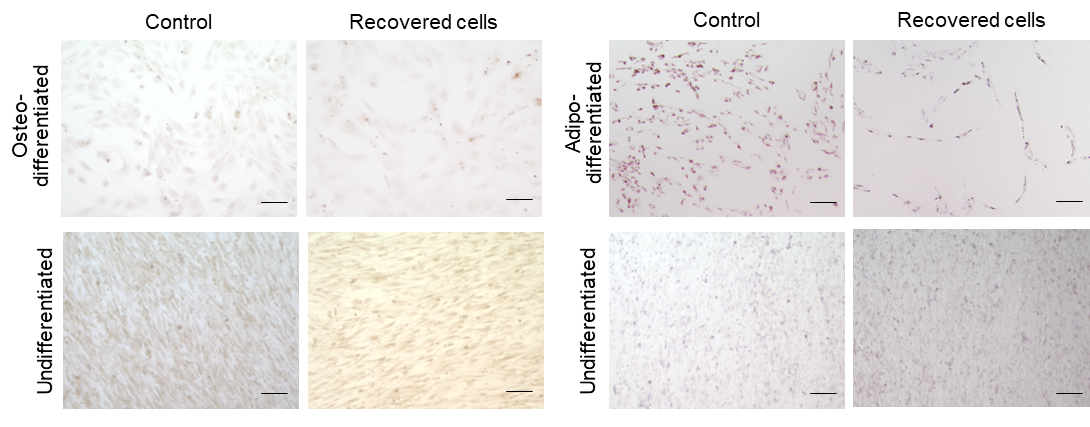
**

**Fig. S10** Differentiation potential of the recovered BMMSCs. Osteogenic and adipogenic differentiation was confirmed via Alizarin Red S and Oil Red O staining. Scale bar: 100 μm.
